# Expanding the FurC (PerR) regulon in *Anabaena* (*Nostoc*) sp. PCC 7120: Genome-wide identification of novel direct targets uncovers FurC participation in central carbon metabolism regulation

**DOI:** 10.1371/journal.pone.0289761

**Published:** 2023-08-07

**Authors:** Cristina Sarasa-Buisan, Jorge Guío, M. Luisa Peleato, María F. Fillat, Emma Sevilla

**Affiliations:** Departamento de Bioquímica y Biología Molecular y Celular and Institute for Biocomputation and Physics of Complex Systems, Universidad de Zaragoza, Zaragoza, Spain; BOKU: Universitat fur Bodenkultur Wien, AUSTRIA

## Abstract

FurC (PerR, Peroxide Response Regulator) from *Anabaena* sp. PCC 7120 (also known as *Nostoc* sp. PCC 7120) is a master regulator engaged in the modulation of relevant processes including the response to oxidative stress, photosynthesis and nitrogen fixation. Previous differential gene expression analysis of a *furC*-overexpressing strain (EB2770FurC) allowed the inference of a putative FurC DNA-binding consensus sequence. In the present work, more data concerning the regulon of the FurC protein were obtained through the searching of the putative FurC-box in the whole *Anabaena* sp. PCC 7120 genome. The total amount of novel FurC-DNA binding sites found in the promoter regions of genes with known function was validated by electrophoretic mobility shift assays (EMSA) identifying 22 new FurC targets. Some of these identified targets display relevant roles in nitrogen fixation (*hetR* and *hgdC*) and carbon assimilation processes (*cmpR*, *glgP1* and *opcA*), suggesting that FurC could be an additional player for the harmonization of carbon and nitrogen metabolisms. Moreover, differential gene expression of a selection of newly identified FurC targets was measured by Real Time RT-PCR in the *furC*-overexpressing strain (EB2770FurC) comparing to *Anabaena* sp. PCC 7120 revealing that in most of these cases FurC could act as a transcriptional activator.

## Introduction

FUR (Ferric Uptake Regulator) proteins are prokaryotic metalloregulators mainly involved in the control of metal homeostasis and the concerted response to oxidative stress [1]. The *Anabaena* sp. PCC 7120 genome holds three paralogs belonging to the FUR family that are called FurA, FurB and FurC. FurC was firstly identified as a member of the family because it showed the distinctive features of FUR proteins [2] and it was later proposed that FurC was the PerR paralog in *Anabaena* sp. PCC 7120 [3]. This hypothesis was based on the fact that FurC was able to bind the regions upstream from genes encoding key proteins involved in the response to oxidative stress such as PrxA (Peroxiredoxin A) and SrxA (Sulfiredoxin A) and that displayed *in vitro* the canonical derepression mechanism of PerR proteins which is based on the metal catalyzed oxidation (MCO) process. In this process, FurC was inactivated by oxidation with Fe^2+^ under aerobic conditions hampering its DNA binding activity. Such inactivation can be prevented by previous incubation with excess of manganese. Later on it was demonstrated that the MCO performed by FurC happened *in vivo* in *Anabaena* sp. PCC 7120 cells allowing the derepression of antioxidant enzymes to face oxidative stress situations [4]. More recently, it was shown that in fact the irreversible inactivation of FurC/PerR by Fe^2+^ in aerobic conditions goes along with the incorporation of an oxygen atom into its structure as its observed for other PerR orthologs [5, 6]. This study also disclosed the FurC/PerR metal binding and oligomerization properties describing an additional layer of regulation of the protein based on cysteine-based reversible oxidation, increasing the complexity of its regulatory mechanism. Apart from its role in regulation of oxidative stress response genes, it was reported that FurC encompassed the regulation of other crucial processes in *Anabaena* sp. PCC 7120 such as the maintenance of the photosynthetic metabolism [4]. The overexpression of FurC in *Anabaena* sp. PCC 7120 led to changes in the composition and efficiency of the photosynthetic machinery and novel FurC targets were described such as the major thylakoid membrane protease FtsH which degrades D1 proteins in photoinhibition processes involved in PSII recycling [4].

Subsequently, differential gene expression analyses were carried out comparing the *furC*-overexpressing strain (EB2770FurC) transcriptional profile with that of the wild type strain (*Anabaena* sp. PCC 7120) under standard and diazotrophic growth conditions. These analyses evidenced that FurC was a global regulator involved in the control of a wide variety of processes including photosynthesis, iron homeostasis, energy metabolism, heterocyst development and nitrogen fixation [7]. In this study, several genes related to nitrogen metabolism were found to be directly regulated by FurC such as *hetZ*, *asr1734*, *hepC*, *alr4973-75*, *nifH2*, *nifHDK*, *xisHI* and *rbrA* [7]. Interestingly, the *furC*-overexpressing strain showed an impairment of heterocyst differentiation under combined nitrogen deficient conditions and serious difficulties to grow under diazotrophic conditions [7]. In addition, it was observed that FurC could also act as transcriptional activator displaying a dual role in its transcriptional regulation. The vast amount of novel direct targets of FurC retrieved from the differential gene expression analyses allowed the inference of a putative FurC-box yielding the FurC DNA-binding consensus sequence 5´- CAAAATCATAACGACTTTG-3´ [7].

Our previous studies suggested that FurC could be acting as a global player in *Anabaena* sp. PCC 7120. In the present work, we try to expand the direct regulatory network exerted by FurC and decipher new metabolic pathways modulated by this protein. To go further in the knowledge of the FurC regulon, an *in silico* identification of novel FurC-DNA binding sites was performed scanning the total *Anabaena* sp. PCC 7120 genome sequence with a more restrictive version of the putative FurC-box defined previously [7]. Furthermore, the potential FurC-regulated genes were analyzed by EMSA to identify actual direct targets of FurC. Additionally, Real Time RT-PCR experiments were performed comparing the transcriptional levels of the potential FurC targets in the *furC* overexpressing strain EB2770FurC and the wild-type *Anabaena* to study the potential effect of the FurC regulation. Among others, this study unveiled several FurC direct targets displaying important roles in nitrogen and carbon metabolisms.

## Materials and methods

### Bioinformatic prediction of FurC putative binding sites

A FurC position-weight-matrix was generated by MEME (Multiple Em for Motif Elicitation) using the six FurC-DNA binding sequences previously identified in the tested promoter regions of *prxA*, *ftsH*, *furC*, *srxA*, *nifH2* and *ahpC* genes [7] using the default parameters. This FurC-matrix was used as the input for the software Find Individual Motif Ocurrences (FIMO) (http://meme-suite.org/tools/fimo) [8], to identify the best matches within the *Anabaena* sp. PCC 7120 genome (*Nostoc* sp. PCC 7120 = FACHB-418) using a p-value <1x10^-5^ as cut-off. Predicted FurC boxes were mapped to annotated CoDing Sequences (CDS) from GenBank assembly (GCA_000009705.1; Sept 2020). Genes with predicted FurC boxes within -1000 bp upstream (intergenic regions) and +50 bp downstream with respect to its start codon were selected as potential FurC targets. In the cases where occurrences were found in the intergenic region of divergently transcribed genes, the gene with closer CDS to the putative FurC-box was chosen as the potential regulated gene. Gene symbol and protein function of the associated genes of the predicted FurC binding sites were annotated according to the databases KEGG (https://www.genome.jp/kegg/) and the information available in literature.

### Electrophoretic Mobility Shift Assays (EMSA)

Promoter regions used in the analyses consisted of 250–350 bp DNA fragments containing the predicted FurC binding sites and were obtained by PCR, using the *Anabaena* sp. PCC 7120 genome as template and the primers included in S1 Table. EMSA analyses were performed with a FurC protein purified as previously described [7]. Reactions for EMSA analyses were performed by mixing increasing concentrations of purified FurC in a final volume of 20 °l with 50 ng of DNA fragments in a binding buffer containing 10 mM Bis Tris-HCl, pH 7.5, 40 mM KCl, 0.1 mg/ml BSA, 1 mM DTT (1,4-dithiothreitol), 100 °M of MnCl_2_ and 5% (v/v) glycerol. To assess the specificity of the assay 50 ng of a 150 bp-internal fragment of the gene *pkn22* was used as a non-specific competitor The resulting mixture was incubated for 30 min at room temperature. Afterwards DNA fragments were added to the mixture and held for 30 min at room temperature. In all cases the incubated mixtures were loaded into a non-denaturing 6% polyacrylamide gel. Both gel and running buffer included 100 °M MnCl_2_. Gels were stained with SYBR ® Safe (Invitrogen) and visualized in a GelDoc 2000 device (Bio-Rad). In all assays, the specific binding to the previously recognized FurC target, *hetZ*, was used as a positive control.

### Cultures and RNA extraction

The RNA samples used for the most of the comparative gene expression experiments performed in this work were extracted and purified as in Sarasa-Buisan et al. 2022 [7]. Briefly, total RNA was prepared from *Anabaena* sp. PCC 7120 and EB2770FurC strains. The EB2770FurC strain is a *furC*-overexpressing strain that contains the pAM2770FurC plasmid harboring the *furC* gene downstream the copper-inducible *petE* (plastocyanin) promoter [4]. Three independent cultures of each strain were set up by diluting a pre-inoculum from late exponential phase until an OD750 of 0.4 in a final volume of 100 ml in BG11. Cultures were grown in Erlenmeyer flasks on an orbital shaker at 130 rpm and 28°C under a continuous light regime of 30 μmol photons·m^-2^·s^-1^ and samples were collected after 48 hours (OD_750_ = 0.6–0.7). RNA was extracted and purified from 25 ml of each culture following the method described in Sarasa-Buisan et al. 2022 [7]. In the experiments for measuring *hetR* expression overtime, the RNA samples used were extracted from cultures set up as stated above and collecting the samples at 1, 6, 11, 24 and 48 hours after the beginning of the experiment. To study differential expression under oxidative stress conditions, RNA samples were obtained from cultures set up as stated above but incubating the cell suspensions at an OD_750_ of 0.4 in the presence and absence of 250 μM H_2_O_2._ The samples were collected after 1 hour of H_2_O_2_ treatment. RNA extraction and purification of all experiments were performed from 25 ml of each culture following the method described in Sarasa-Buisan et al. 2022 [7].

### Real time RT-PCR assays

The pool of cDNA was synthesized by reverse-transcription of 2 °g of total RNA using SuperScript retrotranscriptase (Invitrogen) following the manufacturer’s conditions. Real time PCR was performed using the ViiA™ 7 Real-Time PCR System (Applied Biosystems). Each reaction was set up by mixing 12.5 °l of SYBR Green PCR Master Mix with 0.4 μl of 25 °M primer mixture and 10 ng of cDNA template in a final volume of 30 °l. Amplification was performed at 60°C. Negative controls with no cDNA were included. The sequences of specific primers of selected genes are defined in S1 Table. Transcript levels of target genes were normalized to those of the housekeeping gene *rnpB* measured with the same samples [9]. Relative quantification was performed according to the comparative Ct method (ΔΔCt Method) [10]. The minimum fold-change threshold was set up to ± 1.5 fold.

## Results

### Prediction of novel FurC putative binding sites in the Anabaena sp. PCC 7120 genome

In order to identify potential FurC binding sites, the whole genome of *Anabaena* sp. PCC 7120 was scanned by a program trained on previously identified sites. The program, FIMO, compares the sequence of a candidate binding site against the nucleotide tendencies at each position of sequences within the training set. Searching of FurC binding sites was performed with a 19-bp FurC-matrix built through MEME software using as input the six sequences closest to a motif found in promoter regions of genes putatively regulated by FurC (*prxA*, *ftsH*, *furC*, *srxA*, *nifH2* and *ahpC*) [7]. The expression of these genes changed in response to *furC* overexpression, and their promoter sequences bound FurC in gel shift experiments [3, 4, 7]. The motifs used as a training set as well as the logo built by MEME software are shown in S1 Fig. FIMO analyses found a total of 210 motif occurrences in the genome of *Anabaena* sp. PCC 7120 (p-value < 1x10^-5^) (S2 Table). From the total prediction 55 motif occurrences were found in the putative promoter regions of *Anabaena* sp. PCC 7120 genome defined as -1000 upstream (intergenic region) and +50 bp downstream the translation start site including two motif occurrences with the closest CDSs corresponding to pseudogenes, whereas interestingly 155 motif occurrences were found outside this region, being 151 located inside the coding regions of genes and 4 located in intergenic convergent regions. The location with respect to the translation start sites of closest CDS is shown in S2 Table. The novel predicted FurC boxes found in the putative promoter regions of genes from *Anabaena* sp. PCC 7120 with a *p*-value lower than a 10^−5^ threshold are listed in Table 1. In those cases where motif occurrences were found in the intergenic region of divergently transcribed genes, the gene with closer CDS to the putative FurC-box was chosen as the potentially regulated gene for further analyses. All FurC binding sites located in the promoter regions were found in those from chromosomal genes except for two predicted binding sites located in the promoter region of two genes located in the α plasmid of *Anabaena* sp. PCC 7120, namely *asr7140* and *asr7090* both encoding both hypothetical proteins. In addition, all the potential FurC targets presented a single predicted FurC binding site in their promoter regions with the exception of *all2761* which codes for a hypothetical protein that presents two overlapping predicted FurC boxes. To locate the predicted FurC binding-sites in the promoter regions, the relative position of FurC boxes to the translational start codon (ATG) is shown in Table 1.

**Table 1:** Novel predicted FurC-DNA binding sites by FIMO and its potentially regulated gene.

ORF	Gene/role^a^	p-value	Predicted FurC-box^b^	Distance to ATG^c^
*alr5164*	***degQ3****;* deg protease	4.17x10^-9^	CGTAGTCATTATGAATTTA	9
*all0737*	***ntrC;*** NADPH-thioredoxin reductase C	7.84x10^-8^	CGAACTCATAACGACTACG	11
*all0465*	hypothetical protein	1.28x10^-7^	CAAAGTCACTATAATTTTG	91
*all0473*	***zupT; zinc transporter***	2.19x10^-7^	CATAGTCATTTCAACTATG	34
*alr0359*	hypothetical protein	3.25x10^-7^	CAAAATCATAATGATATAG	144
*all0879*	zinc-binding alcohol dehydrogenase family protein	9.22x10^-7^	AATACTAATTACAACTTTG	71
*asr2041*	putative XRE family transcriptional regulator	1.07x10^-6^	CGTAATCATAACAATATAA	185
*alr0760*	hypothetical protein	1.39x10^-6^	CACAGTCATAACGACATAG	9
*all5105*	hypothetical protein	1.58 x10^-6^	TGAAATCATTACCACTTTG	122
*alr4334*	***pheA***; prephenate dehydratase	1.64x10^-6^	CAATGTCATTATGTTTTTA	9
*all1123*	Orange Carotenoid Protein NTD-paralog	1.76x10^-6^	CGCAATCATTATGACTTTT	9
*all1272*	***glgP1***; glycogen phosphorylase	1.85x10^-6^	CAAAGTCATGGCAATTTTA	81
*all4018*	***opcA***; glucosa-6-phosphate dehydrogenase assembly protein	2.04x10^-6^	GGAAGTCATAATAATTGTG	9
*alr3281*	putative transcriptional regulator	2.11x10^-6^	AGTAGTCATACTAACATTA	9
*alr1604*	hypothetical protein	2.26x10^-6^	CGTAATTATTACTATTTTG	52
*all5305*	hypothetical protein	2.39x10^-6^	CCTAATCATTATGTTTTTG	9
*all5346*	***hgdC***: heterocyst specific ABC-transporter	2.53x10^-6^	AAAATTCATATTGATTTTG	9
*all2761*	hypothetical protein	2.94x10^-6^	AGAAATCTTAATAATAATG	3
*alr1794*	hypothetical protein	3.06x10^-6^	CGTACTCATTACTCCTATG	3
*all2611*	hypothetical protein	3.15 x10^-6^	CAAAGTAATAATATTTTTG	5
*alr2137*	two-component system, NarL family, sensor kinase	3.25x10^-6^	CGTAATGTTTACGATTATG	134
*alr4559*	WD-40 repeat-protein	3.36x10^-6^	CTTACTCATTATTATTATG	169
*alr4124*	S-adenosylmethionine synthetase	3.50x10^-6^	TGAAATCATAACAATCTTG	191
*all4441*	probable glycosyltransferase	3.85x10^-6^	ATTACTCATAACGACTTTC	9
*alr4601*	hypothetical protein	3.85x10^-6^	CAAAGTCCTAATGTTAATG	1
*asr7140*	hypothetical protein	3.91x10^-6^	AAAAATCATAAACATTTTG	49
*asl0095*	hypothetical protein	4.18x10^-6^	CAAATACATAATGAAATTG	7
*all2761*	hypothetical protein	5.14x10^-6^	CTTAATAATAATGACTTTA	9
*alr0252*	Na^+^/H^+^ antiporter	5.42x10^-6^	AAAAATCATAACGATTCAA	189
*all0862*	***cmpR***; low CO_2_-responsive LysR family transcriptional regulator	5.53x10^-6^	CATACTTATAACGAGAATG	191
*alr0309*	hypothetical protein	5.79x10^-6^	ATAACTCATAATAAAAATA	44
*all0067*	cob(I)alamin adenosyltransferase	5.83x10^-6^	CAAAATCAAATTGATTATA	69
*alr0159*	probable glycosyltransferase	5.96x10^-6^	AATAATAATTACGATTTTT	101
*alr3809*	***carB***; carbamoyl phosphate synthase	6.33x10^-6^	AAAAGTCAAAATGATCATA	128
*asr2369*	hypothetical protein	6.33x10^-6^	AAAAGTCTTAACGAAAGTG	124
*asr3992*	***psbZ***; photosystem II protein Z	6.42x10^-6^	AGAAGTCAAAATGACCATA	9
*alr2339*	***hetR***; heterocyst differentiation protein	6.85x10^-6^	AGTAGTCATAATGGCTTAA	838
*all3797*	***fas1*;** fasciclin domain-containing protein	7.05x10^-6^	AAATCTCATAACTAATTTG	115
*all0945*	***sdhB****;* succinate dehydrogenase iron-sulfur protein subunit	8.25x10^-6^	CATAACCATTAAAATTTTA	155
*all3578*	***dnaENI***; DNA polymerase subunit III	8.25x10^-6^	TGTAATCACTATGAATATG	37
*all2038*	hypothetical protein	8.38x10^-6^	CGTACTAATTAAAAATTTG	9
*alr4548*	***psbD***; photosystem II D2 protein	8.79x10^-6^	CGAACTTATAAAAACATTG	13
*alr2325*	***ancrpB***; cAMP receptor protein transcriptional regulator	8.89x10^-6^	ATAAGTAATAATAACTATG	170
*alr0840*	unknown protein	9.26x10^-6^	CATACTTAGAATAATTTTG	57
*all3292*	hypothetical protein	9.60x10^-6^	AGTAATCTTTATTACTTTG	129
*asr7090*	hypothetical protein	9.65x10^-6^	AGAAGTCTTAACGACAAGA	172
*alr2822*	hep island protein	9.90x10^-6^	CAAAATCTTTAAAAATTTA	286

^a^ Gene name and description according to the Kyoto Encyclopedia of Genes and Genomes (https://www.kegg.jp) and information available in literature.

^b^ FurC binding sites obtained by FIMO in the promoter regions of genes from *Anabaena* sp. PCC 7120 genome defined as -1000 bp upstream (intergenic region) and +50 bp downstream with respect to the start codon.

^c^ Distance to ATG defined as number of nucleotides from the translational start site to the closest end position of the predicted FurC box.

### Determination of FurC binding to predicted sites

**Fig 1:**
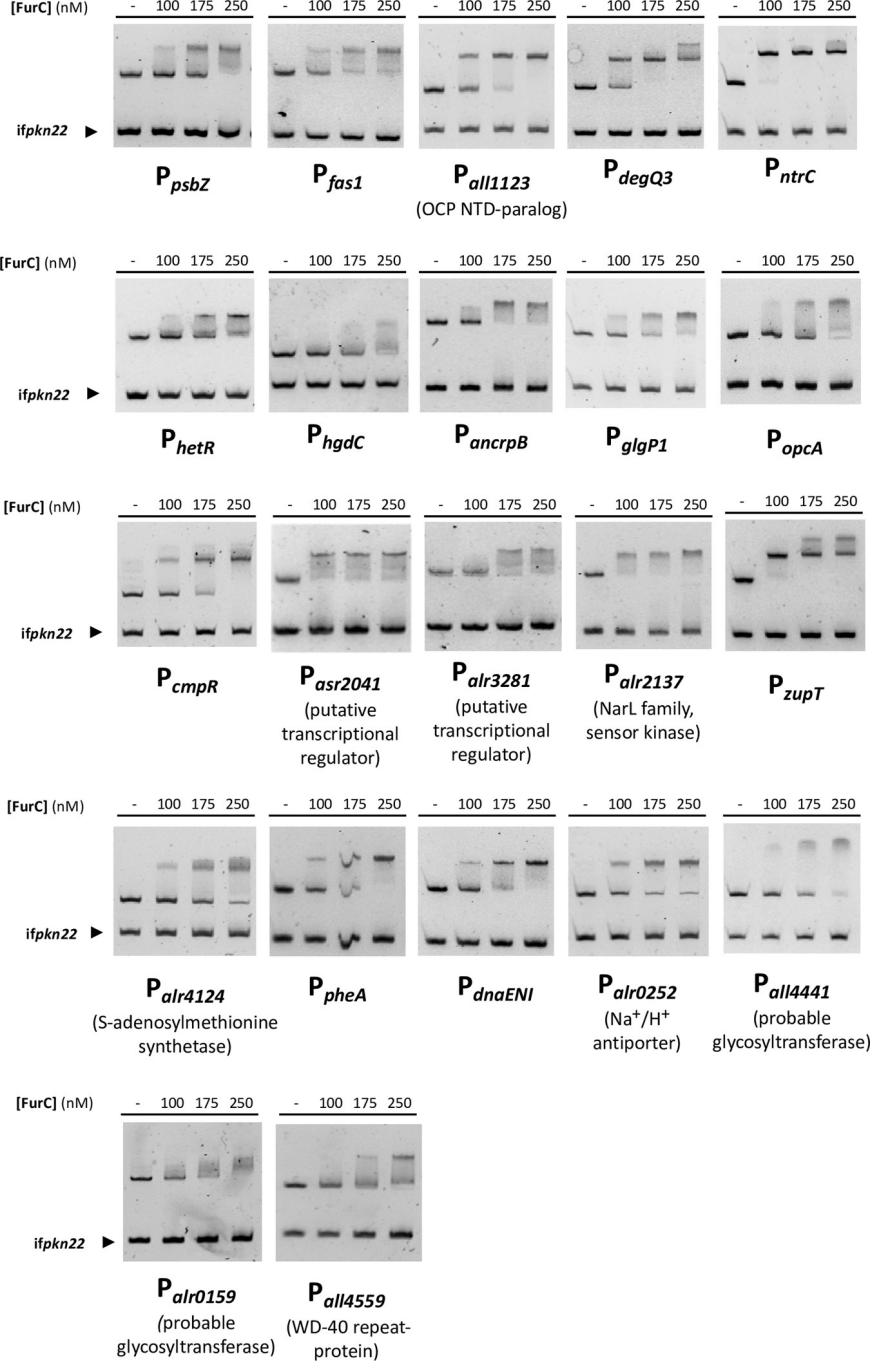
Validation of the prediction performed by EMSA assays. The ability of FurC to bind the promoter regions of selected genes was tested. DNA fragments free or mixed with increasing concentrations of recombinant FurC (nM) were separated by 6% PAGE. An internal fragment of the *pkn22* gene was used as non-specific competitor DNA.

The potential binding of FurC to the predicted sites was addressed by performing EMSA assays with all the promoter regions of novel potential FurC targets that presented a predicted FurC-box listed in Table 1 excluding those from genes encoding hypothetical proteins. The EMSA assays showed that among the selected 28 novel potential FurC direct targets, 22 of them were confirmed as actual direct targets of FurC (Fig 1). FurC direct targets were organized in functional categories (Table 2). FurC predicted binding sites were located upstream of genes that take part in a wide range of cellular process such as photosynthesis and photoprotection, oxidative stress, nitrogen metabolism and heterocyst differentiation, central carbon metabolism, zinc homeostasis, amino acid metabolism, as well as regulatory functions, among others. Several EMSA-confirmed FurC predicted binding sites were associated with genes encoding proteins related to photosynthesis and photoprotection. This is consistent with the involvement of FurC in those processes that was described in our previous reported results [4]. Genes as *psbZ* (*asr3992*) encoding a PSII structural subunit, *fas1* (*all3797*), an ortholog of *fas1* from *Anabaena* sp. strain L31 that seems to be involved in the response of this cyanobacterium to UV-B radiation stress [11] and *all1123*, one of the paralogs to the N-terminal domain of the Orange Carotenoid Protein (OCP) present in *Anabaena* sp. PCC 7120 [12] were direct targets of FurC (Fig 1 and Table 2). OCP proteins interact with the antennas regulating the amount of excitation energy that reaches the reaction centers [13]. However, All1123 seems not to be displaying this particular role in photoprotection, instead it has been suggested that might work as a carotenoid-transport protein [12] or being involved in the response to UV-B stress [14]. Finally, FurC bound to the *alr5164* promoter (Fig 1 and Table 2) that correspond to one of the three DegQ protease homologues in *Anabaena* sp. PCC 7120 [15]. In *Synechocystis*, DegQ is a protease implicated in the degradation of the PsbO subunit from the PSII [16] and its expression increases under high light conditions [17]. Another EMSA-confirmed FurC predicted binding site corresponded to that located usptream *ntrC* (*alr0737*) encoding the NADPH-tiorredoxin reductase C that plays a fundamental role in oxidative stress management and participates in the thermotolerance and regulation of N metabolism [18, 19].

**Table 2:** Novel putative FurC direct targets found by the genome FurC box search and validated by EMSA.

ORF	Gene/role^a^	EMSA^b^
Photosynthesis and photoprotection		
***all0067***	cob(I)alamin adenosyltransferase	**-**
***all1123***	Orange Carotenoid Protein NTD-paralog	**+**
***all3797***	***fas1;*** fasciclin domain-containing protein	**+**
***alr4548***	***psbD***; photosystem II D2 protein	**-**
***alr5164***	***degQ3;*** deg protease	**+**
***asr3992***	***psbZ;*** photosystem II protein Z	**+**
Oxidative stress response		
***all0737***	***ntrC;*** NADPH-thioredoxin reductase C	**+**
Nitrogen metabolism and Heterocyst differentiation		
***all5346***	***hgdC;*** heterocyst specific ABC-transporter	**+**
***alr2325***	***ancrpB***; cAMP receptor protein transcriptional regulator	**+**
***alr2822***	hep island protein	**-**
***alr2339***	***hetR;*** heterocyst differentiation protein	**+**
Central carbon metabolism		
***all0862***	***cmpR***; low CO_2_-responsive LysR family transcriptional regulator	**+**
***all0945***	***sdhB****;* succinate dehydrogenase iron-sulfur protein subunit	**-**
***all1272***	***glgP1***; glycogen phosphorylase	**+**
***all4018***	***opcA***; glucose-6-phosphate dehydrogenase assembly protein	**+**
***alr3809***	***carB***; carbamoyl phosphate synthase	**-**
Regulatory functions		
***alr2137***	two-component system, NarL family, sensor kinase	**+**
***asr2041***	putative XRE family transcriptional regulator	**+**
***alr3281***	putative transcriptional regulator	**+**
Zinc homeostasis		
***all0473***	***zupT; zinc transporter***	**+**
Amino acid metabolism		
***alr4124***	S-adenosylmethionine synthetase	**+**
***alr4334***	***pheA***; prephenate dehydratase	**+**
Other		
***all0879***	zinc-binding alcohol dehydrogenase family protein	**-**
***all3578***	***dnaENI***; DNA polymerase subunit III	**+**
***all4441***	probable glycosyltransferase	**+**
***alr0159***	probable glycosyltransferase	**+**
***alr0252***	Na^+^/H^+^ antiporter	**+**
***alr4559***	WD-40 repeat-protein	**+**

^a^ Gene name and description according to the Kyoto Encyclopedia of Genes and Genomes (https://www.kegg.jp) and information available in literature. Genes annotated with a specific name are shown in bold.

^b^ EMSA validation of potential FurC targets showing the result as +/-.

Regarding to the “Nitrogen metabolism and heterocyst differentiation” category, FurC was capable of binding to the promoter regions of genes encoding proteins with fundamental roles in the regulation of heterocyst development and nitrogen fixation such as the heterocyst specific ABC transporter, HgdC and, remarkably, the master regulator of the heterocyst differentiation HetR (Fig 1 and Table 2). HgdC takes part of an ABC transporter called Tol-HgdBC located in the cell wall of heterocysts. This transporter participates in the formation of the proper heterocyst glycolipid layer (HGL) in the heterocyst envelope [20]. Interestingly, FurC was able to bind to the *hetR* promoter. HetR is a key regulator in the heterocyst development and it is required for a suitable pattern formation [21–23]. EMSA assays also indicated that *ancrpB* (*alr2325*) is a direct target of FurC. *Anabaena* sp. PCC 7120 possesses two cAMP receptor proteins called AnCrpA and AnCrpB [24]. AnCrpA regulates the expression of gene clusters relevant in nitrogen fixation processes such as *nifB*, *hglE* or *coxBII* [24]. However, the role of AnCrpB is not that clear because this transcriptional regulator was found to be upregulated in response to rehydration situations [25], presumably controlling the expression of genes involved in the early response to rehydration although it also regulates the expression of some genes related to the response to nitrogen depletion [25].

Moreover, some genes related to carbohydrate metabolism and carbon fixation were verified as FurC direct targets (Fig 1 and Table 2). This is the case of *glgP1* (*all1272*) the glycogen phosphorylase involved in glycogen degradation processes, *opcA*, the enzyme that activates the glucose 6-phosphate dehydrogenase (G6PDH) in the heterocyst and provides reducing power for the nitrogenase and *cmpR (all0862)*, encoding a LysR type transcriptional regulator that activates the Cmp bicarbonate transport systems in response to low carbon conditions (see below). Another FurC putative binding sites corroborated *in vitro* were upstream of genes displaying regulatory functions such as the putative transcriptional regulators Asr2041 and Alr3281 and the Alr2137 sensor kinase (Fig 1 and Table 2). Noticeably, another direct target associated with the predicted FurC binding site was the zinc transporter ZupT (*all0473*) (Fig 1 and Table 2). This gene is the ortholog of *zupT* from *Escherichia coli* which encodes a low affinity zinc importer responsible for zinc uptake during zinc-sufficient conditions [26, 27]. Other confirmed FurC targets were genes related to amino acid metabolism, such as *alr4124* and *pheA* and genes encoding proteins displaying other functions such as *dnaENI* which encodes a DNA polymerase subunit III, *alr0252*, an Na^+^/H^+^ antiporter, two probable glycosyltransferases *all4441* and *alr0159*, and *all4559* encoding a WD-40 repeat protein (Fig 1 and Table 2).

### Differential gene expression analysis of newly identified FurC targets in a furC-overexpressing strain versus Anabaena sp. PCC 7120

**Fig 2:**
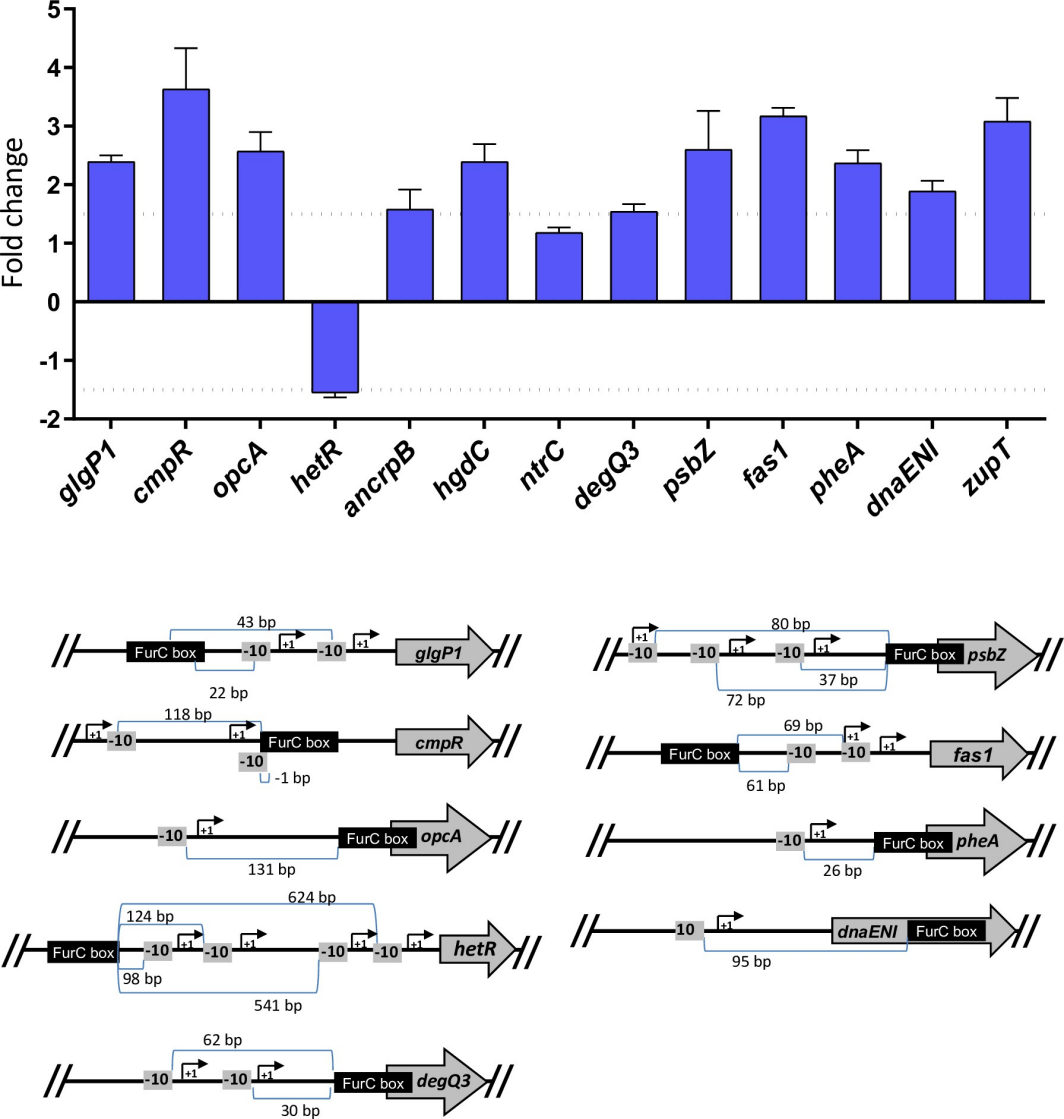
Transcriptional analysis of the novel FurC direct targets in a *furC*-overexpressing strain and promoter analysis. (A) Influence of *furC* overexpression on the mRNA levels of selected genes (see text for choice of genes). Relative Real Time RT-PCR was used. Values are expressed as fold change (EB2770FurC vs. wild-type strain) and correspond to the average of three biological and three technical replicates; the standard deviation is indicated. (B) Graphical representation of the FurC box location in the promoter region of selected genes. The relative positions of FurC boxes related to -10 elements and the tsps reported in Mitschke at al. 2011 [29] are shown. -10 elements are represented as -10 and the distance in bp is indicated. In the case of *hetR* tsps are retrieved from Buikema and Haselkorn. 2001 [30] and Mitschke et al. 2011 [29].

Transcriptional analyses were conducted to compare the expression levels of the selected novel FurC direct targets in a *furC*-overexpressing strain (EB2770FurC) versus the wild type strain *Anabaena* sp. PCC 7120. The *furC*-overexpressing strain was used instead of a *furC* knockout mutant because previous attempts to obtain the knockout strain performed by our group and other groups were unsuccessful [3, 4, 7]. Twelve genes were chosen because they were annotated with a specific gene name (*glgP1*, *cmpR*, *opcA*, *hetR*, *ancrpB*, *hgdC*, *ntrC*, *degQ3*, *psbZ*, *fas1*, *pheA*, *dnaENI* and *zupT*). All the tested genes except for *ntrC* showed altered expression in the *furC*-overexpressing strain thus confirming their direct regulation by FurC (Fig 2). Interestingly, most of the selected novel FurC direct targets were overexpressed in the EB2770FurC strain with respect to the wild type *Anabaena*. Only *hetR* was slightly downregulated in the *furC*-overexpressing strain (Fig 2). In the case of *glgP1* and *fas1* the FurC box is located upstream of the predicted -10 elements as well as in the previously reported FurC direct targets *asr1734*, *ftsH*, *hepC*, *rbrA* and *all3914* [7]. In these cases, FurC could act as a transcriptional activator. On the other hand, transcriptional repressors can act hampering the binding of the RNA polymerase to the -10 element directly or binding downstream the -10 elements [28]. This category would include the previously reported FurC-regulated genes *prxA*, *srxA*, *CGT3* and *psbAIV* [7]. Curiously, a large number of sites reported here (*opcA*, *degQ3*, *pheA*, *psbZ*) sit directly on the start codon of the genes. In these cases, although they are found upregulated in the *furC*-overexpressing strain, the mechanism exerted by FurC on these genes is still unknown.

### FurC could act as transcriptional activator following the canonical MCO mechanism

**Fig 3:**
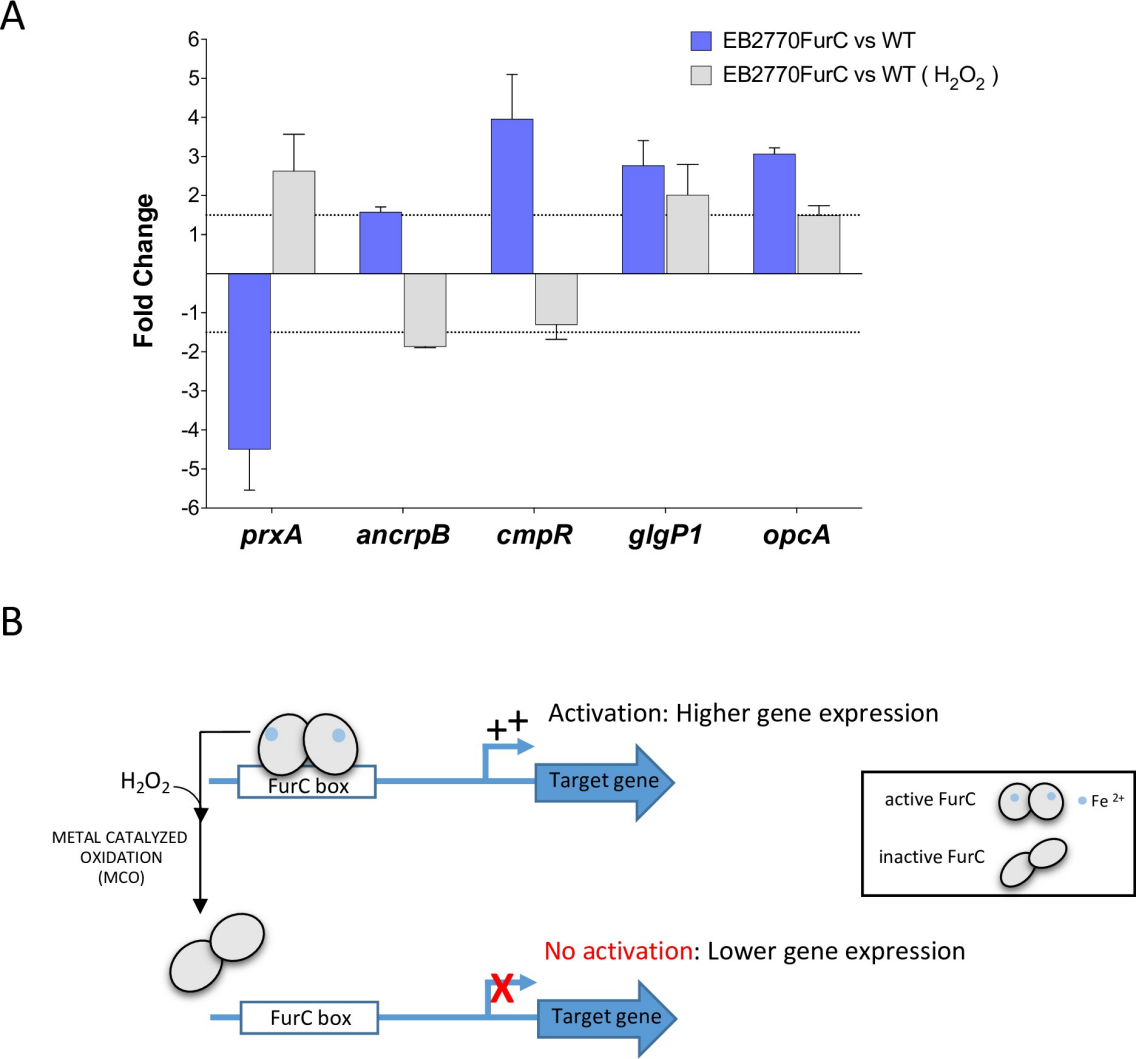
Study of the FurC-mediated activation of gene expression in response to oxidative stress (A) Relative transcription (EB2770FurC vs. *Anabaena* sp. PCC 7120) of selected genes in presence and absence of the oxidative challenge imposed by H_2_O_2_ measured by Real Time RT-PCR. Values are expressed as fold change (EB2770FurC vs. wild-type strain) and correspond to the average of three biological and three technical replicates; the standard deviation is indicated. (B) Graphical representation of the FurC-mediated peroxide sensitive mechanism for activation of gene expression. Under oxidative conditions caused by H_2_O_2_ under iron repletion metal catalyzed oxidation of FurC is produced, ceasing the activation of its targets that occurs in absence of oxidative conditions.

A big amount of new FurC targets described in the present and our previous work (Sarasa-Buisan et al. 2022) seem to be activated by FurC since their expression increases in the *furC*- overexpressing strain. In one previous work we demonstrated that when FurC acts as transcriptional repressor, this protein follows the classical MCO mechanism of PerR proteins *in vivo* [4]. This fact means that when FurC is bound to iron in the presence of hydrogen peroxide, FurC is oxidized and then released from its target promoters allowing the transcription. In the present work we wanted to analyze the mechanism of action in those occasions in which FurC acts as transcriptional activator. The expression of *ancrpB*, *glgP1*, *cmpR* and *opcA* FurC activated genes and *prxA* as a control of FurC repressed gene was analyzed after the culture exposure to the presence of 250 μM of hydrogen peroxide for 1 hour. The results are included in Fig 3. As we previously described [4], the expression of *prxA* is repressed under standard conditions whereas in the presence of hydrogen peroxide the expression is derepressed because FurC was released from the *prxA* promoter (Fig 3A). In the activated genes *ancrpB*, *cmpR* and *opcA* in which the expression increases in the *furC*-overexpression strain, the expression decreases in the presence of hydrogen peroxide indicating the releasing of FurC from the promoter and the cease of the activation (Fig 3A and 3B). However, In the case of *glgP1* the expression barely change under oxidative stress conditions (Fig 3A). This phenomenon could be explained by the observation made in the well characterized PerR regulon from *Bacillus subtilis*, in which not all the genes are peroxide regulated, suggesting that PerR protein may exists in distinct Fe or Mn metalated forms that differ in their target selectivity and their sensitivity to oxidation [31].

### HetR expression could be controlled by FurC

**Fig 4:**
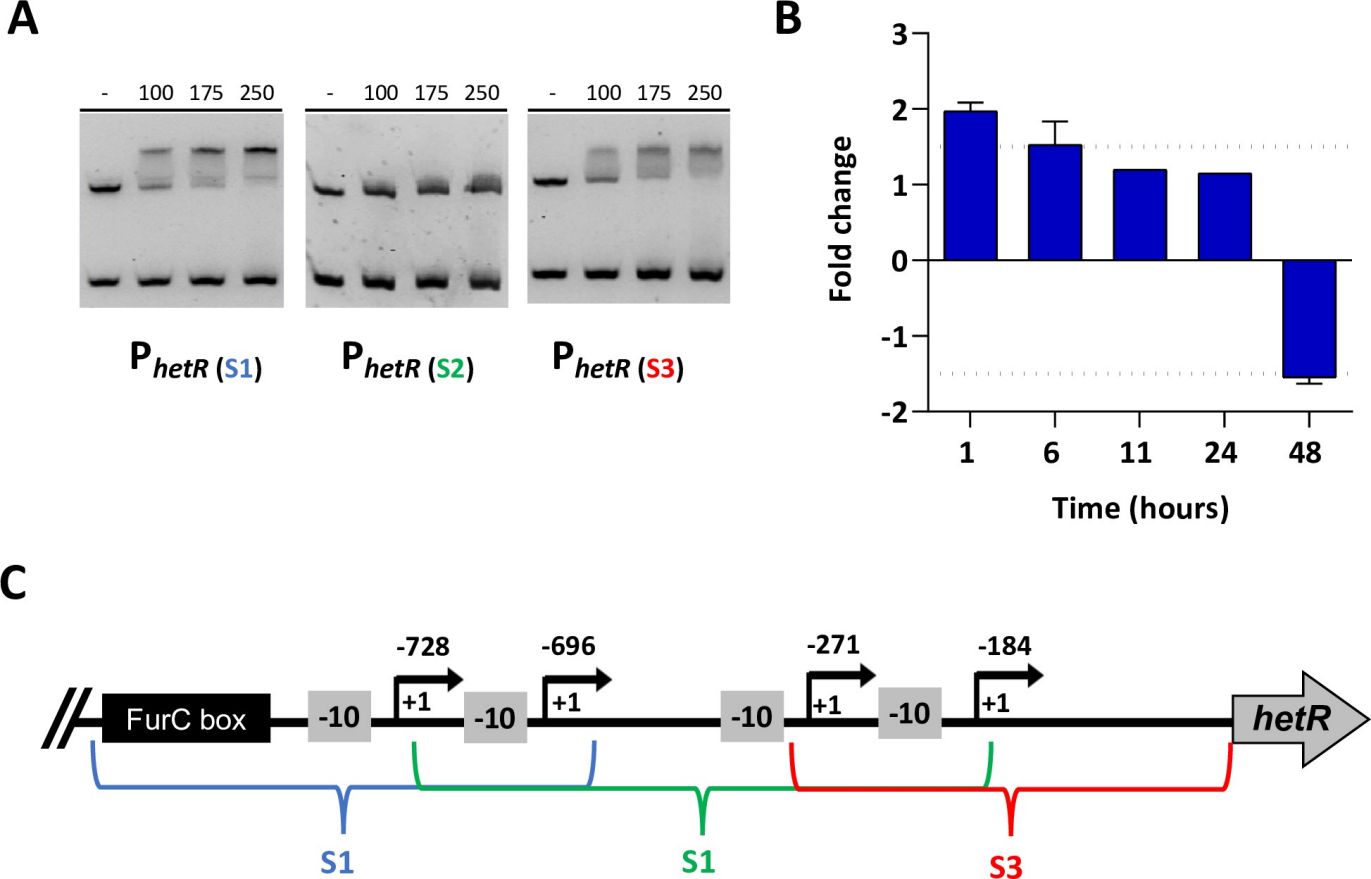
Analysis of *hetR* transcriptional regulation performed by FurC. (A) Electrophoretic mobility shift assays showing the binding of FurC to the *hetR* promoter region. The promoter was separated in three DNA regions named S1, S2 and S3. DNA fragments free or mixed with increasing concentrations of recombinant FurC were separated by 6% PAGE. An internal fragment of the *pkn22* gene was used as non-specific competitor DNA. (B) Relative transcription of *hetR* along the time (1, 6, 11, 24 and 48 h) determined by Real-Time RT-PCR in EB2770FurC cells related to *Anabaena* sp. PCC 7120 ones. Values are expressed as fold change and correspond to the average of three biological and three technical replicates; the SD is indicated. (C) Schematic representation of *hetR* promoter showing the tsps (+1), -10 elements and FurC box.

In the present work we found that FurC was able to bind to the promoter region of *hetR* suggesting a potential regulation mediated by this transcriptional regulator (Fig 1). Since the FurC box was predicted by FIMO software in the distal region of the *hetR* upstream region (S1), this was the region firstly analyzed in EMSA assays, yielding a positive result. Afterwards other regions upstream of *hetR* were also analyzed by EMSA assays with FurC (Fig 4A), indicating binding of FurC to the proximal region as well. The results revealed that FurC was also able to bind the proximal region of the *hetR* promoter (S3) (Fig 4A). If the *hetR* promoter region is analyzed in detail, the FurC box is located upstream of -10 element in the distal S1 region, however the existence of a second FurC binding site in the proximal S3 region makes difficult to understand how FurC is regulating the *hetR* expression (Figs 2B and 4C). Then, the *hetR* expression was analyzed by Real Time RT-PCR in time-course experiments (1, 6, 11, 24 and 48h) in BG11 medium comparing the EB2770FurC strain with the wild type strain *Anabaena* sp. PCC 7120 (Fig 4B). We found that the expression of *hetR* changed along the time in the EB2770FurC strain, being upregulated almost 2-fold after 1h of the beginning of the experiment but downregulated approximately 1.5-fold after 48 h. However, after the first hour, changes in expression are hardly significant. This alteration in the mRNA levels of *hetR* in the EB2770FurC strain suggests that FurC could be regulating the transcription of *hetR in vivo*. However, the reason why the *hetR* expression is first upregulated and subsequently downregulated along the time is unknown. As the genetic background of these strains was identical along the experiment, a possible explanation for this transcriptional change could be that the signals which up to date, have been described to modulate the activity of FurC such as the redox state or metal availability [3, 4, 7] vary across the time in the culture media, leading to changes in the expression profile. Anyway, more experiments must be carried out in the future to clarify this observation.

## Discussion

An accurate prediction of the FurC/PerR regulon in *Anabaena* sp. PCC 7120 is essential for our understanding of the scope of the regulation exerted by this protein. Previous work led to the discovery of novel roles of the Peroxide response Regulator beyond the control of oxidative stress management, including the regulation of the photosynthetic metabolism and the heterocyst differentiation [4, 7]. These studies established the selection of new potential targets on the basis of phenotypic observations and transcriptional analysis in a *furC* overexpressing strain. In the present work an *in silico* approach has allowed us to obtain a global vision of the extent of the FurC regulon. The bioinformatic search for novel FurC binding sites predicted 210 FurC boxes in the whole genome of *Anabaena* sp PCC 7120. Interestingly, although it could be considered striking, a considerable number (151) of them were located in internal positions of the CDSs (S2 Table). However, multiple similar cases have been found in literature for other bacterial transcriptional regulators [32–36]. One is the case of the master regulator of nitrogen metabolism NtcA in *Anabaena* sp. PCC 7120, in which a ChIP-seq analysis identified a high proportion (65%) of NtcA binding regions inside or even downstream of coding regions [36]. More recently, strong pieces of evidence for a meaningful binding of bacterial transcription factors to coding regions have been reported for seven model bacteria, describing three regulatory mechanisms of CDS-bound transcriptional regulators within individual genes, operons, and antisense RNAs [35]. Several possibilities for a specific regulation exerted in these internal sites could be extended for FurC. These possibilities include the potential regulation of internal or antisense transcriptional start sites. This hypothesis would be in agreement with the fact that some of the ORFs that contain the FurC boxes located inside coding regions present either internal or antisense transcriptional start sites [29]. Moreover, a possible role blocking transcription elongation as has been characterized previously for the global transcriptional regulator CodY in *B*. *subtilis* [32] could also be possible. Given the complexity for these cases, the present work centered the attention in the FurC boxes found in the putative promoter regions, although we cannot rule out a possible function of FurC outside these regions. If we focus on the predicted FurC binding sites located in putative promoter sequences, among the 28 potential FurC targets selected for EMSA validation, 22 were shown as direct FurC targets. In addition, 13 of them were proved to have their expression altered in a *furC*-overexpressing strain. Some of these targets displayed key roles in carbon and nitrogen metabolism, photosynthesis and light protection, zinc homeostasis or amino acid metabolism. Carbon and nitrogen metabolisms are tightly coupled in organisms because of a suitable balance of carbon and nitrogen is necessary for optimal growth and proper functioning of the cells [37]. In cyanobacteria, several levels of regulation are superimposed for guarantee the equilibrated uptake and assimilation of nitrogen and carbon sources [37–41]. In *Anabaena* sp. PCC 7120, many aspects related to the regulation and control of carbon and nitrogen homeostasis are not yet well defined. The engagement of FurC in the regulatory network of heterocyst development and nitrogen fixation was previously suggested. However, in the present work we found that another three genes involved in nitrogen metabolism and heterocyst differentiation (*hgdC*, *ancrpB* and *hetR*) are also direct targets of FurC. Firstly, *hgdC* encodes a component of Tol-HgdBC transporter which participates in the formation of HGL layer in heterocyst envelopment [20]. Secondly, *ancrpB* encodes a transcriptional regulator that has been suggested to control the expression of genes related to nitrogen depletion [25]. Surprisingly, FurC was also able to bind to the *hetR* promoter, hierarchically one of the most important regulators in the process of heterocyst development. Despite of its importance, the transcriptional regulation of HetR is barely understood. Until now, NrrA had been described as a direct positive transcriptional regulator of the HetR expression [42, 43]. Besides, FurA (another member of the FUR family in *Anabaena* sp. PCC 7120) was also able to bind to the *hetR* promoter [44]. In addition, some post-transcriptional regulatory mechanisms have been reported for HetR. One of them consists on HetR oligomerization controlled by phosphorylation [45] and the other one defines the interaction with RG(S/T)GR-containing pentapeptides (PatS, PatX and HetN) which act as negative regulators of the HetR activity [46, 47]. In view of this complex panorama, more studies must be carried out to be able to understand the regulation exerted by FurC on the *hetR* expression.

Regarding to carbon metabolism, our results showed that FurC binds *in vitro* to the promoters of three important genes comprised in this functional category named *glgP1*, *cmpR* and *opcA*. In addition, the expression of these genes is upregulated in the EB2770FurC strain. These genes display functions that link both carbon and nitrogen metabolisms which are closely intertwined. GlgP1 is a glycogen phosphorylase which catalyzes the generation of glucose-1-phosphate monomers from the glycogen polymer. *Anabaena* sp. PCC 7120 vegetative cells store carbon belonging to photosynthesis in form of glycogen which is utilized as source of carbon and energy by cyanobacteria during the night. The heterocyst is able to perform photosynthesis (based on PSI) and generate ATP by cyclic photophosphorylation. On the other hand, it has been suggested that vegetative cells could supply carbohydrates (sucrose) to the heterocyst. Some of these carbohydrates derive from glycogen catabolism, indeed NrrA, a nitrogen-regulated response regulator protein, binds to the *glgP1* promoter, controlling glycogen metabolism [48, 49]. On the other hand, CmpR is a transcriptional regulator that activates the transcriptional expression of *alr2877*-*alr2880* gene cluster encoding a Cmp bicarbonate transporter under carbon starvation conditions [50]. The Cmp bicarbonate transporter takes part of the carbon concentrating mechanisms developed by cyanobacteria to concentrate carbon in form of CO_2_ allowing the efficient actuation of Rubisco in carbon fixation [51]. On the other hand, CmpR has also been proposed as a negative regulator of *rbcl* operon (encoding Rubisco) [50]. CmpR expression is also regulated by NtcA which again brings into the light the cross-talk between carbon and nitrogen regulatory networks [50]. Finally, in the present work we found that FurC directly controls the expression of OpcA. The redox protein OpcA acts as allosteric activator of glucose-6-phosphate dehydrogenase (G6PDH) [52, 53]. G6PDH is the initial enzyme of the oxidative pentose phosphate pathway in *Anabaena* sp. PCC 7120 and is essential for nitrogen fixation because this pathway provides NADPH required for the generation of the reducing power for the nitrogenase in heterocysts [54, 55].

Finally, in the present work experiments performed with the *furC*-overexpressing strain in the presence and absence of hydrogen peroxide revealed a potential activation mechanism that could be performed by FurC (Fig 3B). It can be observed that both the repression and the activation mechanisms are both sensitive to peroxide treatment and may take place depending on the MCO mechanism. This fact is very interesting since the MCO mechanism is a complex mechanism that depends on the oxidative stress found in the cell but also on the iron and manganese availability so that FurC could be regulating its target genes integrating these three different signals. In fact, this mechanism of regulation in response to oxidative stress of FurC might be even more intricated, since a new *in vitro* regulatory mechanism for FurC activity was recently reported, evidencing that apart from the MCO, FurC undergoes a reversible oxidation through interdimer disulfide bridges formation under mild oxidant conditions and iron deficiency that reversibly prevents the *in vitro* binding to the DNA [5].

**Fig 5:**
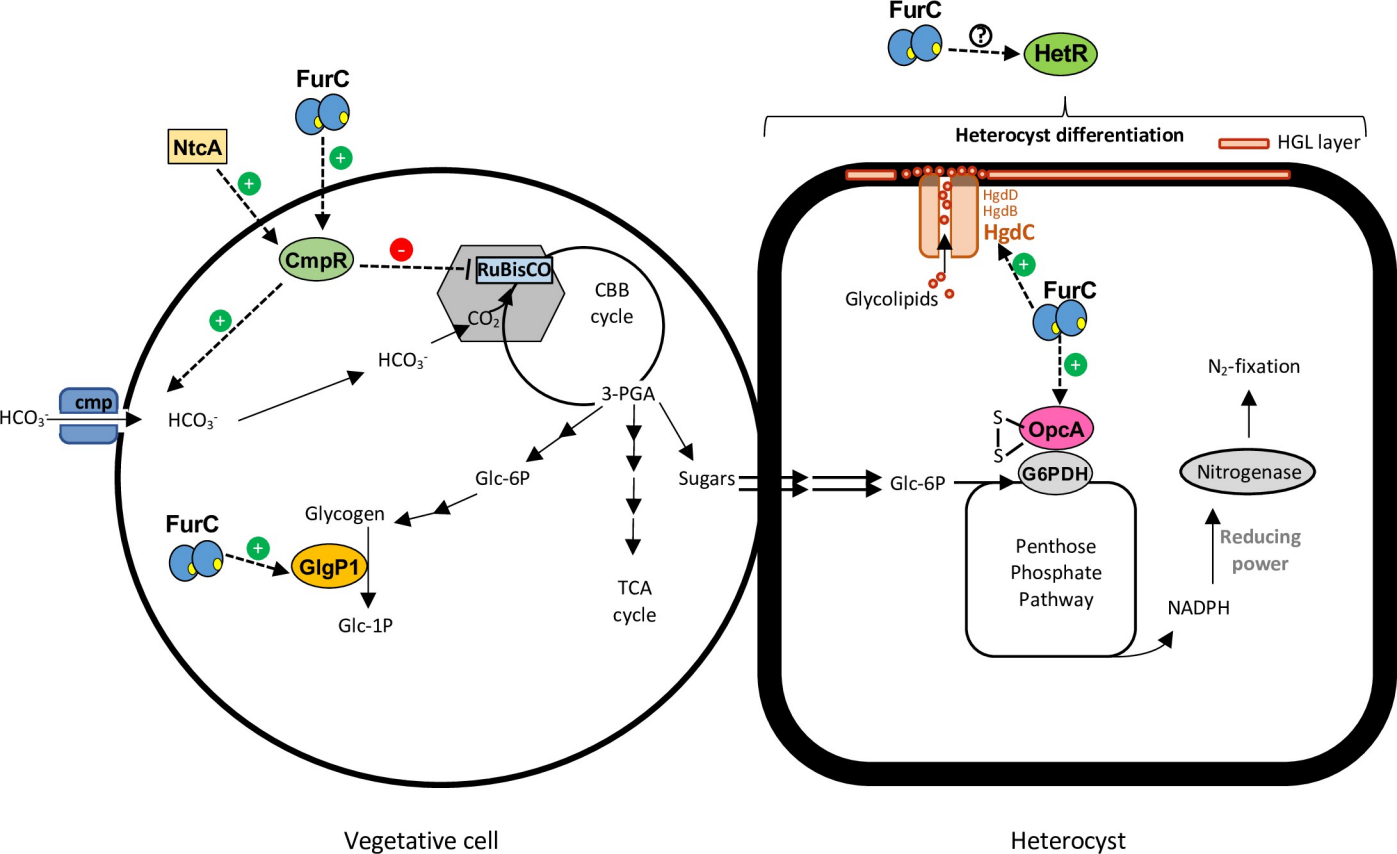
General scheme of the transcriptional regulation performed by FurC of some relevant novel direct target genes involved in the maintenance of nitrogen and carbon homeostasis in *Anabaena* sp. PCC 7120. Vegetative cells as well as heterocyst cells are represented to observe processes regulated by FurC in both types of cells. Dashed lines indicate transcriptional regulation, the symbols (+) and (–) show positive and negative transcriptional regulation respectively. CBB cycle: Calvin–Benson–Bassham cycle; TCA cycle: the tricarboxylic acid cycle; 3-PGA: 3-phosphoglycerate; Glc-6P: glucose-6-phosphate; Glc-1P: glucose-1-phosphate; G6PDH: glucose-6-phosphate dehydrogenase.

In summary, FurC might be modulating different important processes that are essential for the maintenance of nitrogen and carbon homeostasis in *Anabaena* sp. PCC 7120 (Fig 5). Previously, we reported that several genes related to heterocyst differentiation and nitrogen fixation were direct targets of FurC (*hetZ*, *asr1734 hepC*, *nifH*, *xisHI*, *rbrA*…) [7]. In the present work we found that, FurC controls the heterocyst formation process by regulating the HgdC transporter expression which is involved in the heterocyst envelope formation and FurC could be regulating the expression of *hetR*, the master regulator of heterocyst development (Fig 5). Furthermore, FurC regulates the expression of CmpR, a transcriptional regulator that controls the carbon fixation process by triggering the expression of bicarbonate transporter Cmp, which is involved in the uptake of bicarbonate into the cell. Additionally, it has been proposed that CmpR could be controlling the expression of Rubisco, enzyme that performs the carbon fixation in the Calvin Cycle (Fig 5). Moreover, FurC controls the expression of GlpP1 glycogen phosphorylase, key enzyme in the catabolism of glycogen and therefore in carbon homeostasis (Fig 5). Finally, FurC seems to upregulate the expression of OpcA, which activates G6PDH in its oxidized form generating reducing power essential for the functioning of nitrogenase in the nitrogen fixation (Fig 5).

Therefore, this work is another piece of evidence that FurC (PerR) is participating in the regulation of the global metabolism of *Anabaena* sp. PCC 7120, linking the redox state of the cell and the iron and manganese availability with important cellular processes such as photosynthesis, carbon fixation, glycogen catabolism or nitrogen fixation hence confirming the role of FurC (PerR) as a master regulator in *Anabaena* sp. PCC 7120.

## Supporting information

S1 Fig FurC DNA-binding sequences used as input to build the FurC-matrix by MEME software. The genes holding these sequences as well as the *p*-values indicating the degree of similarity with FurC consensus sequence are shown. The logo of the FurC-matrix built by MEME software is included.
(TIF)

S1 Table Oligonucleotides used in this study. (DOCX)

S2 Table Predicted FurC-DNA binding sequences found by FIMO in *Anabaena* sp. PCC 7120 genome. CoDing Sequence (CDS) annotation according to GenBank assembly (GCA_000009705.1; Sept 2020). Rows with predicted FurC boxes within promoter region defined as -1000 bp upstream (intergenic regions) and +50 bp downstream with respect to the start codon are highlighted in green and the putatively regulated gene is depicted. In case when the FurC box was predicted in the promoter region of two divergently transcribed genes, the two genes are shown. Rows with predicted FurC boxes outside promoter regions are colored in grey and location is indicated. Distant to ATG is defined as number of nucleotides from the translational start site to the closest end position of the predicted FurC box.^a^ Previously described FurC-DNA binding sites in Sarasa-Buisan et al., 2022 are shown in blue. ^b^ Motif cooccurrences found downstream coding regions are indicated with (-).
(XLSX)

S1 Raw images (PDF)
