# Supplementary figures and images for: Expanding the FurC (PerR) regulon in Anabaena (Nostoc) sp. PCC 7120: Genome-wide identification of novel direct targets uncovers FurC participation in central carbon metabolism regulation
Source: PLoS One. 2023 Aug 7;18(8):e0289761. doi: 10.1371/journal.pone.0289761 (PMC10406281; doi:10.1371/journal.pone.0289761)

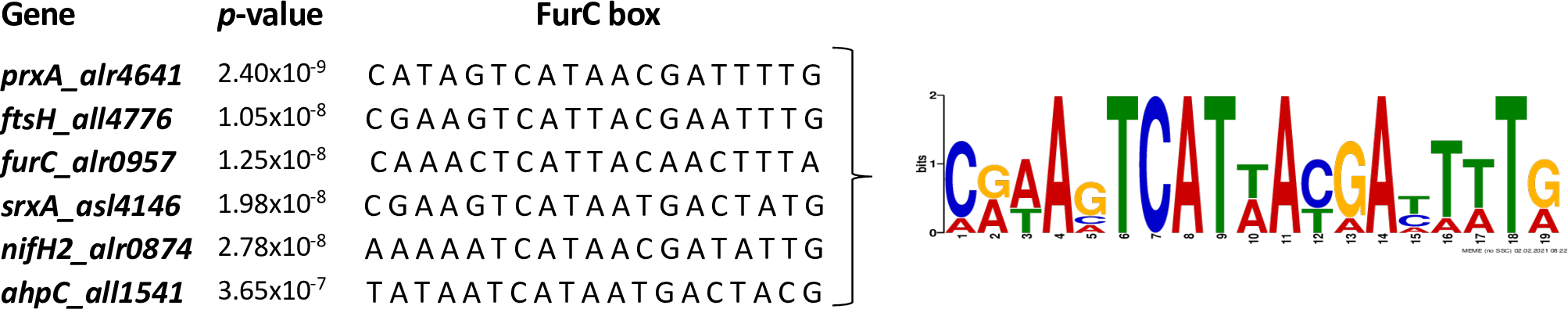

Supplement: S1 Fig — The genes holding these sequences as well as the p-values indicating the degree of similarity with FurC consensus sequence are shown. The logo of the FurC-matrix built by MEME software is included. (TIF) [file pone.0289761.s001.tif]
