# Supplementary material for: Expanding the FurC (PerR) regulon in Anabaena (Nostoc) sp. PCC 7120: Genome-wide identification of novel direct targets uncovers FurC participation in central carbon metabolism regulation
Source: PLoS One. 2023 Aug 7;18(8):e0289761. doi: 10.1371/journal.pone.0289761 (PMC10406281; doi:10.1371/journal.pone.0289761)
Supplement: S1 Table — (DOCX) [file pone.0289761.s002.docx]

| **Supplementary Table S1.** Oligonucleotides used in this study | | | |
| --- | --- | --- | --- |
| Primer | Sequence (5´- 3´) | | Purpose |
|  |  |  | |
| *EMSA* |  |  | |
| Pifpkn_up | AAAGATGAATTACACTGGCG | EMSA unspecific control, internal fragment of *pkn22* | |
| Pifpkn_dw | CTGCAAACTGTGGCAGAATA |  |  |
| Pall0737_up | GGAGTCAGCTGCCGCTTGATG | *all0737 (ntrC)* promoter | |
| Pall0737_dw | GGCCCAGAACCAATAATGAC |  |  |
| Pall0473_up | CTAATCCTTAGTCACACGACAC | *all0473 (zupT)* promoter | |
| Pall0473_dw | GGCATTGATGGGAGACTGAC |  |  |
| Pall0879_up | GGTTTGATGTGAGCCGCAG | *all0879* promoter | |
| Pall0879_dw | CGTAAGGGTTGACGCGGTGC |  |  |
| Palr4334_up | CCGTTGACCTAGATATTCCAG | *alr4334 (pheA)* promoter | |
| Palr4334_dw | GTAGCTTGTTCTGCATAAGTG |  |  |
| Pall1123_up | GTGTAGGGGTGTAGGGATGTAG | *all1123* promoter | |
| Pall1123_dw | GAATAGTGGGATCGTTTGTTTG |  |  |
| Pall1272_up | CAGCCTTCCCTCGGTACATG | *all1272 (glgp1)* promoter | |
| Pall1272_dw | CATTGAATGTGCGAATCGGC |  |  |
| Pall4018_up | CCAGCAGAAGCAGAATTTCTG | *all4018 (opcA)* promoter | |
| Pall4018_dw | CCGCTTCAATTTCGTTTAGCG |  |  |
| Palr4124_up | CAAGATACTCCCTGCTGGAT | *alr4124* promoter | |
| Palr4124_dw | CTAGTCGGGTCTTGTGCCAG |  |  |
| Palr0252_up | GAGTACATATTGGACATTCTCAG | *alr0252* promoter | |
| Palr0252_dw | GACTTCAAATGATGCTTCCATG |  |  |
| Pall0862_up | GTTTGTTCTGATAGCGAGAATAG | *all0862 (cmpR)* promoter | |
| Pall0862_dw | GTATCACTGAATACTTGCACATG |  |  |
| all3797_up | CAAAGCAGACTGCTATACACG | *all3797 (fas1)* promoter | |
| all3797_dw | GCTTGCTTTGATTCACCCTC |  |  |
| alr5164_up | GTTGACAAATTACAAGAGAGCC | *alr5164 (degQ3)* promoter | |
| alr5164_dw | CACCAACCACTAGTAGAGACAG |  |  |
| Pall5346_up | CGCAGATAGCGAACGGGTAG | *alr5346 (hgdC)* promoter | |
| Pall5346_dw | CATCTTGGAAACCAAGTTGC |  |  |
| Pasr2041_up | GATGATAATGGATCACAGGATG | *asr2041* promoter | |
| Pasr2041_dw | TTGACCGATTTTTGGTTGTGC |  |  |
| Palr3281_up | ATCACAGGTGTATCCTTGGG | *alr3281* promoter | |
| Palr3281_dw | CAAAGCGATCGCGCTTCAC |  |  |
| Palr2137_up | GATGTTTGACCAATTGGTGGC | *alr2137* promoter | |
| Palr2137_dw | GAGCGGTAGGCTCAAAAGC |  |  |
| Palr2325_up | CTGGTTAACACTTGTCTAATTAG | *alr2325* promoter | |
| Palr2325_dw | GTGGCTGTACTCAATAAGGG |  |  |
| Pall3578_up | GCCACAAAAGCTTACAATGGTG | *all3578 (dnaENI)* promoter | |
| Pall3578_dw | TAGCTGACTAGCCCCATCCAG |  |  |
| Pall0067_up | GCCAAAGAGTATGAGTCGGG | *all0067* promoter | |
| Pall0067_dw | GATAGAATAGGTTGTGCCGGG |  |  |
| Palr3809_up | CTCCCCAAGGATTAGTATTGGG | *alr3809 (carB)* promoter | |
| Palr3809_dw | GGGCATAGTCGTTGGGTTGG |  |  |
| Pall2368_dw | CAATCAAGAAGACCTGGGGTG |  |  |
| Pasr3992_up | GATAGTGCGCCATTTGATCTA | *asr3992 (psbZ)* promoter | |
| Pasr3992_dw | CAACACAAGCGCTACCAAAGC |  |  |
| Pall0945_up | CAGATGAAGCTGAAGCTCATTTG | *all0945 (sdhB)* promoter | |
| Pall0945_dw | CTTCCATTGCTACCAACCAAA |  |  |
| Palr2822_up | CAAGTTCTCTTGGTTACATTAGG | *alr2822* promoter | |
| Palr2822_dw | GGCGGTAGCTGGTTTAATCTTC |  |  |
| Pall4441_up | CGCCGGCATATTGTTTAAATTGC | *all4441* promoter | |
| Pall4441_dw | CCCGTAGGTTGGTATGACTAC |  |  |
| alr4548-1 | GCGTGTAGCCGATGCTCAAC | *alr4548 (psbD)* promoter | |
| alr4548-2 | CAGTCGTCTAGTACGTCAAAC |  |  |
| PhetR1-up | GGGAAAGTCCTTGTAGGTTAC | *alr2339 (hetR)* promoter distal region | |
| PhetR1-dw | CGCTTGATCAGATCGATG |  |  |
| PhetR2-up | GTCTATAATTTTCCCTCCAG | *alr2339 (hetR)* promoter medium region | |
| PhetR2-dw | CTAATAAGTAACCTACAAG |  |  |
| PhetR3-up | AACCCTTATGACAAAGGAC | *alr2339 (hetR)* promoter proximal region | |
| PhetR3-dw | CTGGAGGGAAAATTATAG |  |  |
| Palr0159_fw | GCGATACTCAATTTATTCATAC | *alr0159* promoter region | |
| Palr0159_rev | CAAGAGGTTAATATTCCCAGG |  |  |
| Pall4559_fw | GCACTCAAGTAAAAATCACAG | *all4559* promoter region | |
| Pall4559_rev | CCAACATTGCTCATAATATACC |  |  |
| PhetZ_dw | GCGTTTAGTTTATCCGCAAA | *alr0099 (hetZ)* promoter | |
| PhetZ_dw | CTCAAGCATTGTTGTAGCCG |  |  |
|  |  |  | |
| *Real Time-PCR* | | | |
| all1272_fw | AAGTCACGGCTTACCACATGAA | *all1272 (glgp1)* | |
| all1272_rev | CTCAACCCCTCTTCTTGAATTAACA |  |  |
| all0862_fw | CGCCGAATTCAGCATTCTG | *all0862 (cmpR)* | |
| all0862_rev | CGATGGGTAAACCATGTACCAA |  |  |
| all4018_fw | CCCCAGAATTGCTGATGAAATC | *all4018 (opcA)* | |
| all4018_rev | CAATGGGAAACAGGGCAATAA |  |  |
| alr5164_fw | CCTCTATTGCGTCGATTTTTCG | *alr5164 (degQ)* | |
| alr5164_rev | CGAACCTGTACCCCTCTCAATT |  |  |
| asr3992_fw | GTGTTGGTGTCTTTTGTCCTAGTTGT | *asr3992 (psbZ)* | |
| asr3992_rev | ACCAAGCCAAAGGAGCTTTTT |  |  |
| all3797_fw | GAGCCTCCCTATAACCGTGGTA | *all3797 (fas1)* | |
| all3797_rev | TGTTGCGGGTTGGGTTTC |  |  |
| alr4334_fw | TTTTACATCCCTATTCCAGCATTG | *alr4334* (*pheA*) | |
| alr4334_rev | ACAGGCACAACAGCTAATTGAGTT |  |  |
| all3578_fw | ACATCTACACCCACCCAAACG | *all3578* (*dnaeNI*) | |
| all3578_rev | TCTGCGCCACATTCCTTACA |  |  |
| all0473_fw | CATTTTCCTTAATAGTCCCTGGTACTG | *all0473 (zupT)* | |
| all0473_rev | GACCATAATTAAAGCGGCATTGA |  |  |
| alr2325_up | GGCGCACCTGTGGAGATG | *alr2325 (ancrpB)* | |
| alr2325_dw | CCAAAAAAATCACCCCTTCCTA |  |  |
| 5346qPCR_fw | TCCACGTTTCTCCGCATCA | *all5346 (hgdC)* | |
| 5346qPCR_fw | GATCAGCACCTGGTTTAAGAGTGA |  |  |
| all0737_fw | TCGGCTTGTGCAATCTGTGA | *all0737 (ntrC)* | |
| all0737_rev | GAGTCGCCAGCACCAATCA |  |  |
| RT_hetR fw | CGCTATGCGAGCCTTAGAAGA | *alr2339 (hetR)* | |
| RT_hetR rv | CAGTTCCTGCATGGCTTCATC |  |  |
| rnpB_for | AGCGGAACTGGTAAAAGACCA | *rnpB* housekeeping | |
| rnpB_rev | GAGAGGTACTGGCTCGGTAAAC |  |  |
