# Supplementary material for: Expanding the FurC (PerR) regulon in Anabaena (Nostoc) sp. PCC 7120: Genome-wide identification of novel direct targets uncovers FurC participation in central carbon metabolism regulation
Source: PLoS One. 2023 Aug 7;18(8):e0289761. doi: 10.1371/journal.pone.0289761 (PMC10406281; doi:10.1371/journal.pone.0289761)
Supplement: S1 Raw images — (PDF) [file pone.0289761.s004.pdf]

Images of uncropped gels from EMSA results presented in Fig 1

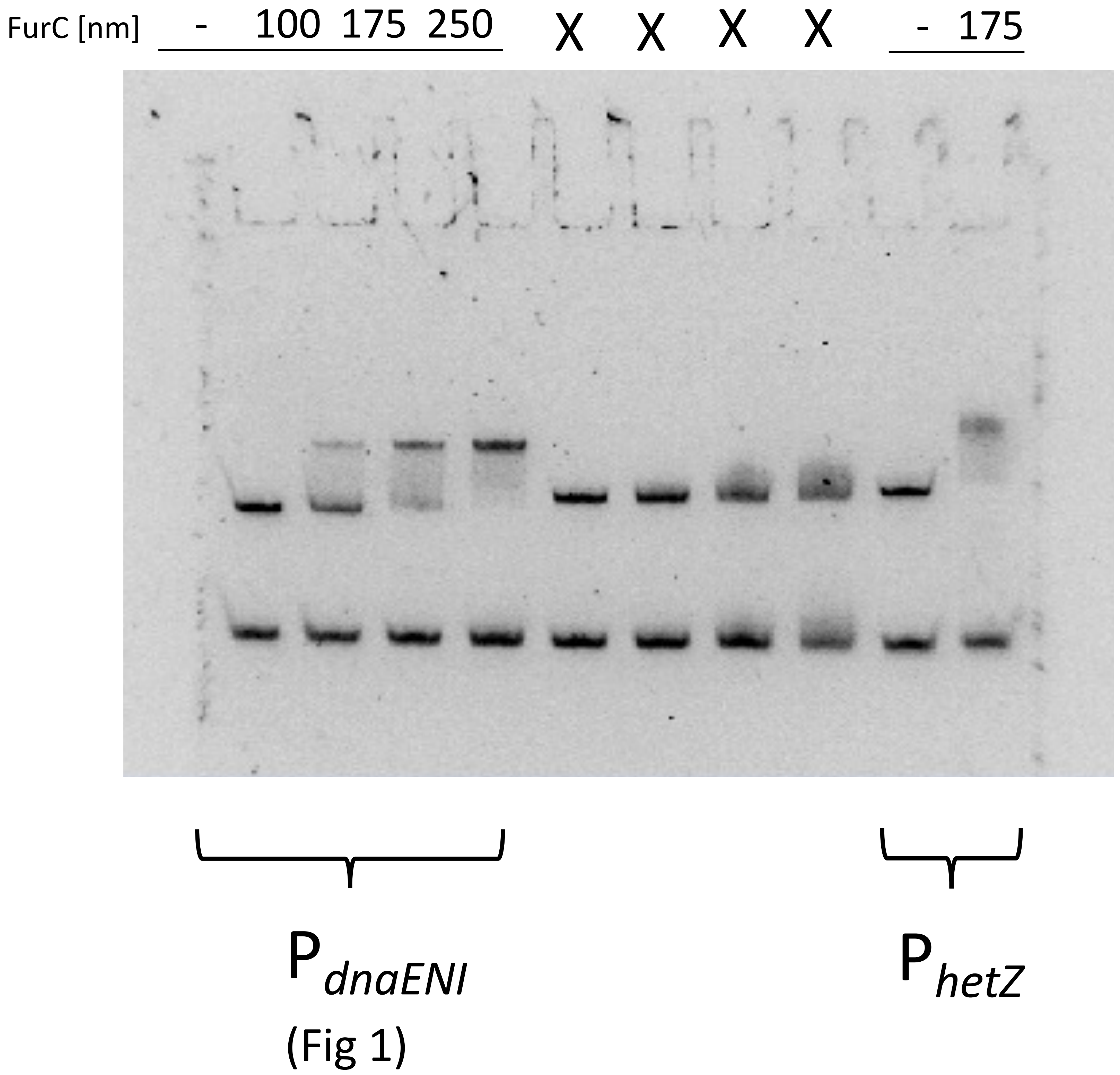
FurC [nm]
- 100 175 250
X X X X
- 175
PdnaENI
(Fig 1)
PhetZ

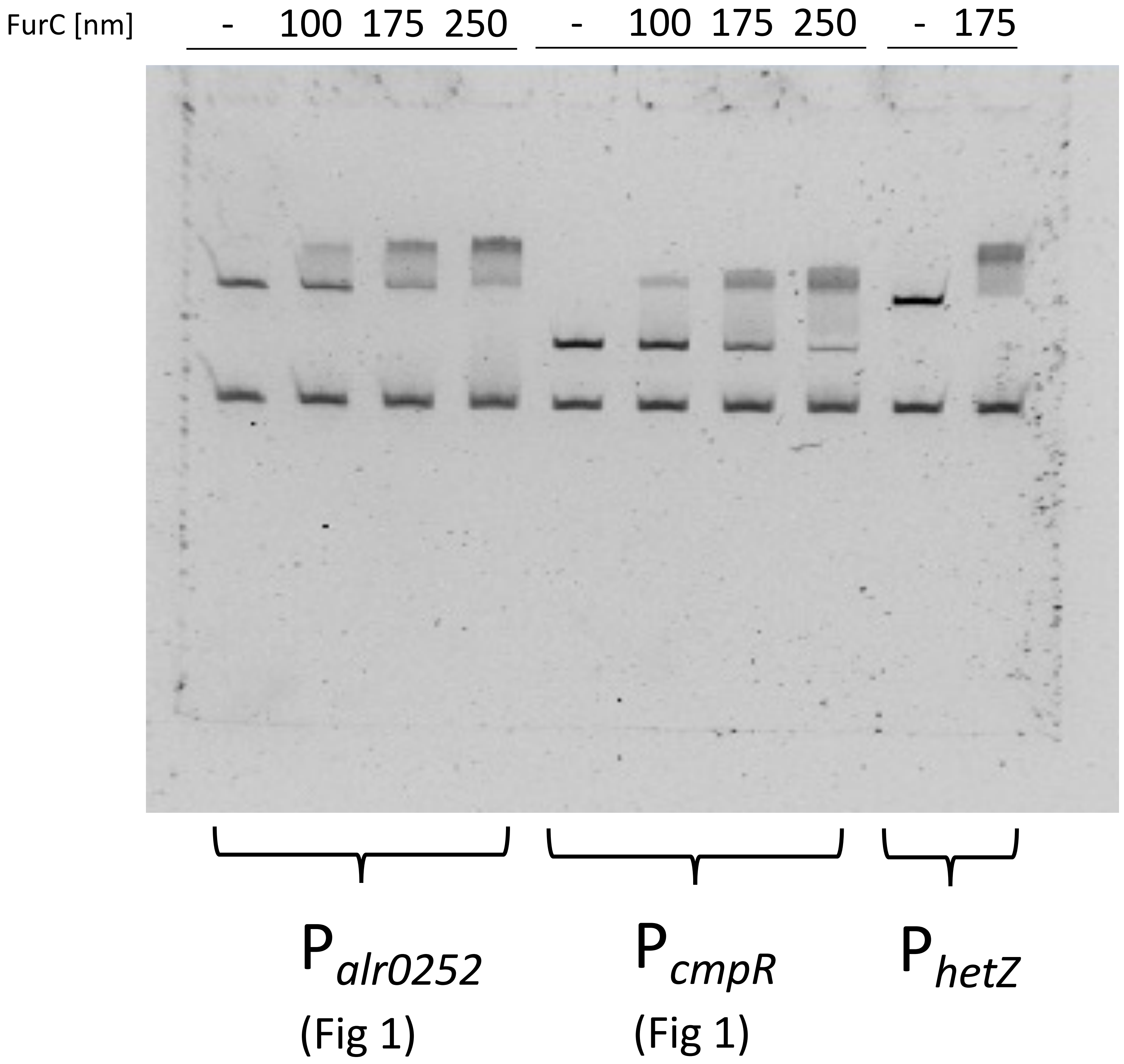
FurC [nm]
- 100 175 250
- 100 175 250
- 175
Palr0252
(Fig 1)
PcmpR
(Fig 1)
PhetZ

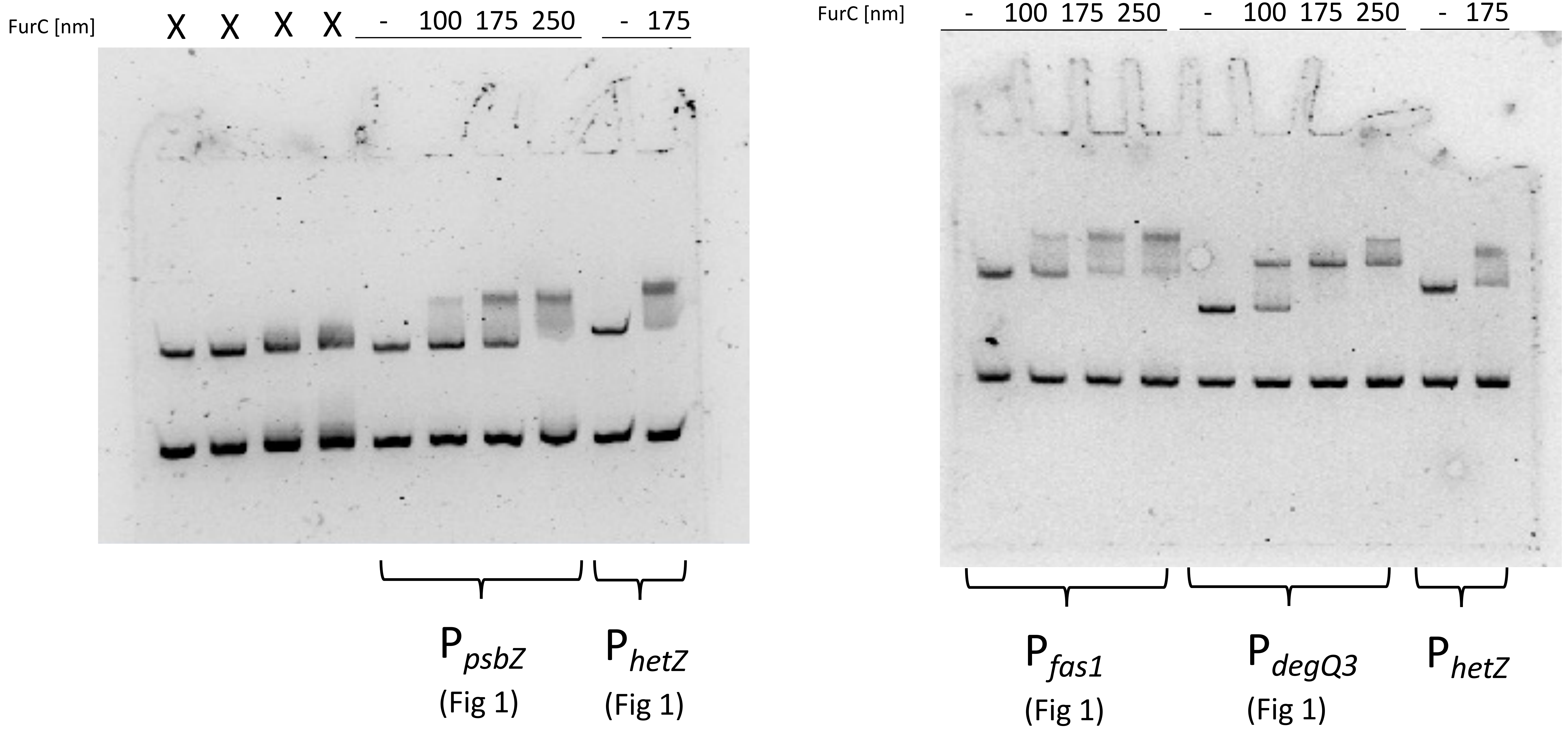

FurC [nm]
X X X X
- 100 175 250
- 175
P_{psbZ}
(Fig 1)
P_{hetZ}
(Fig 1)
FurC [nm]
- 100 175 250
- 100 175 250
- 175
P_{fas1}
(Fig 1)
P_{degQ3}
(Fig 1)
P_{hetZ}

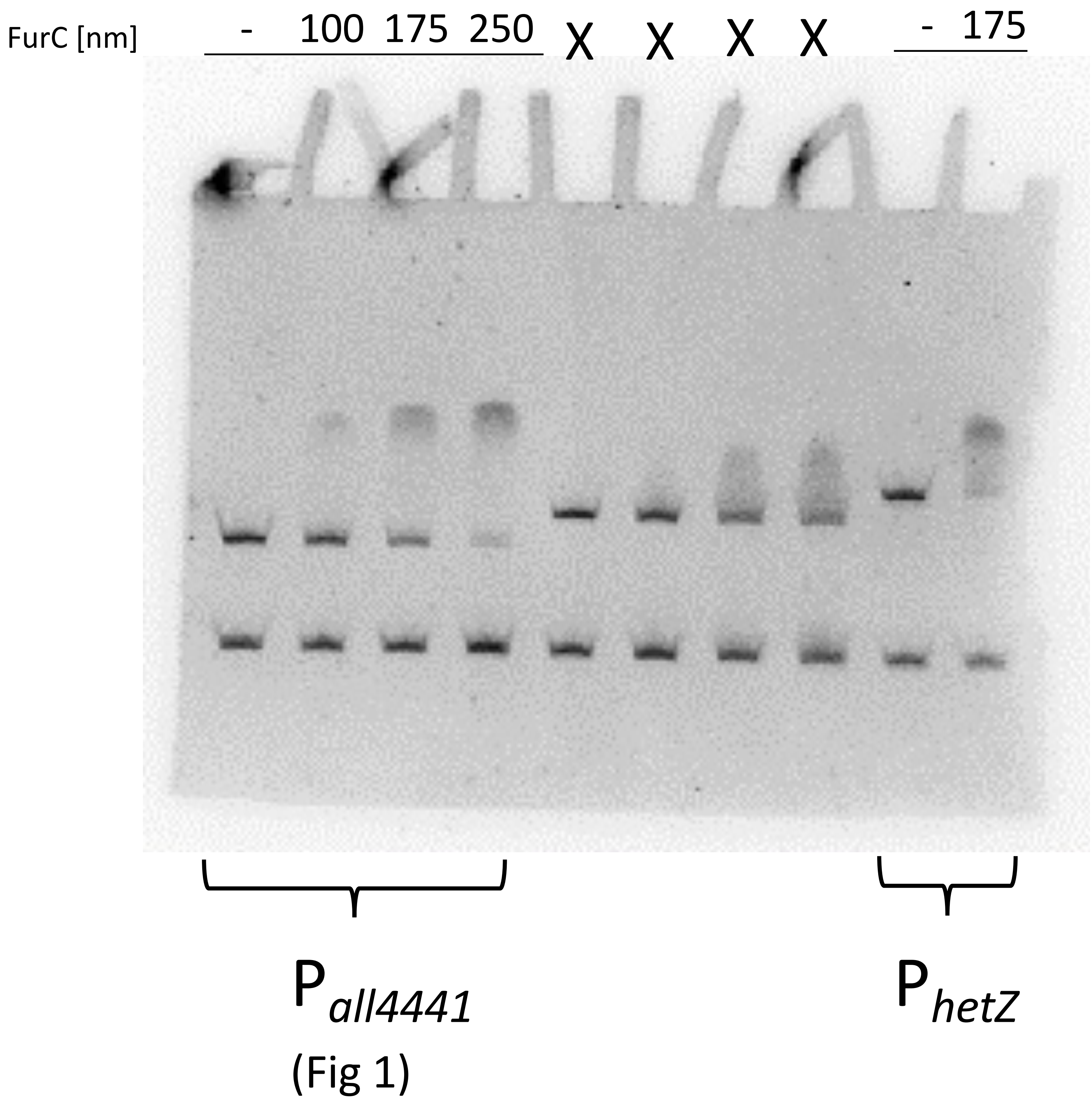
FurC [nm]
- 100 175 250
X X X X
- 175
P all4441
(Fig 1)
P hetZ

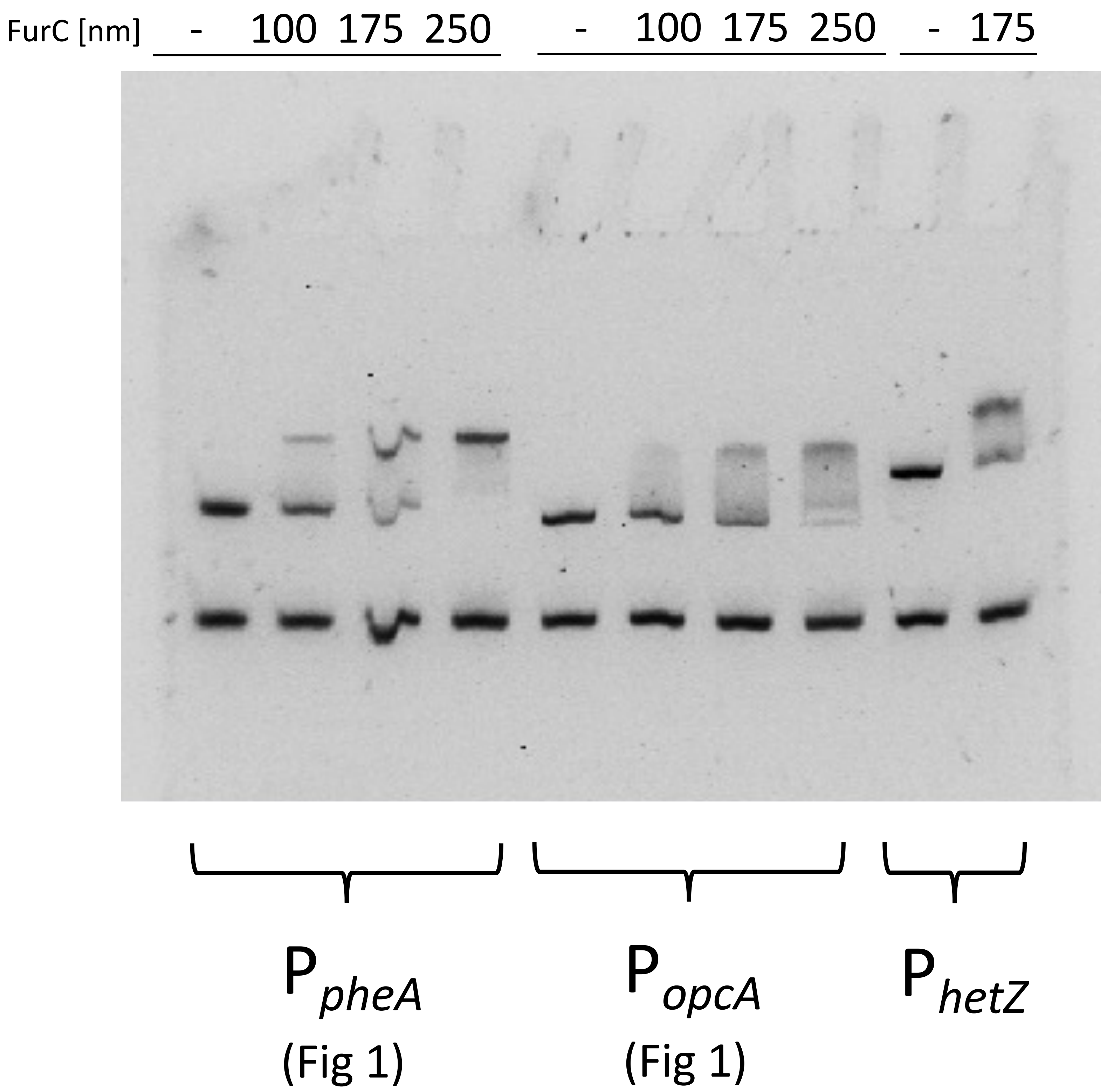
FurC [nm]
- 100 175 250
- 100 175 250
- 175
P pheA
(Fig 1)
P opcA
(Fig 1)
P hetZ

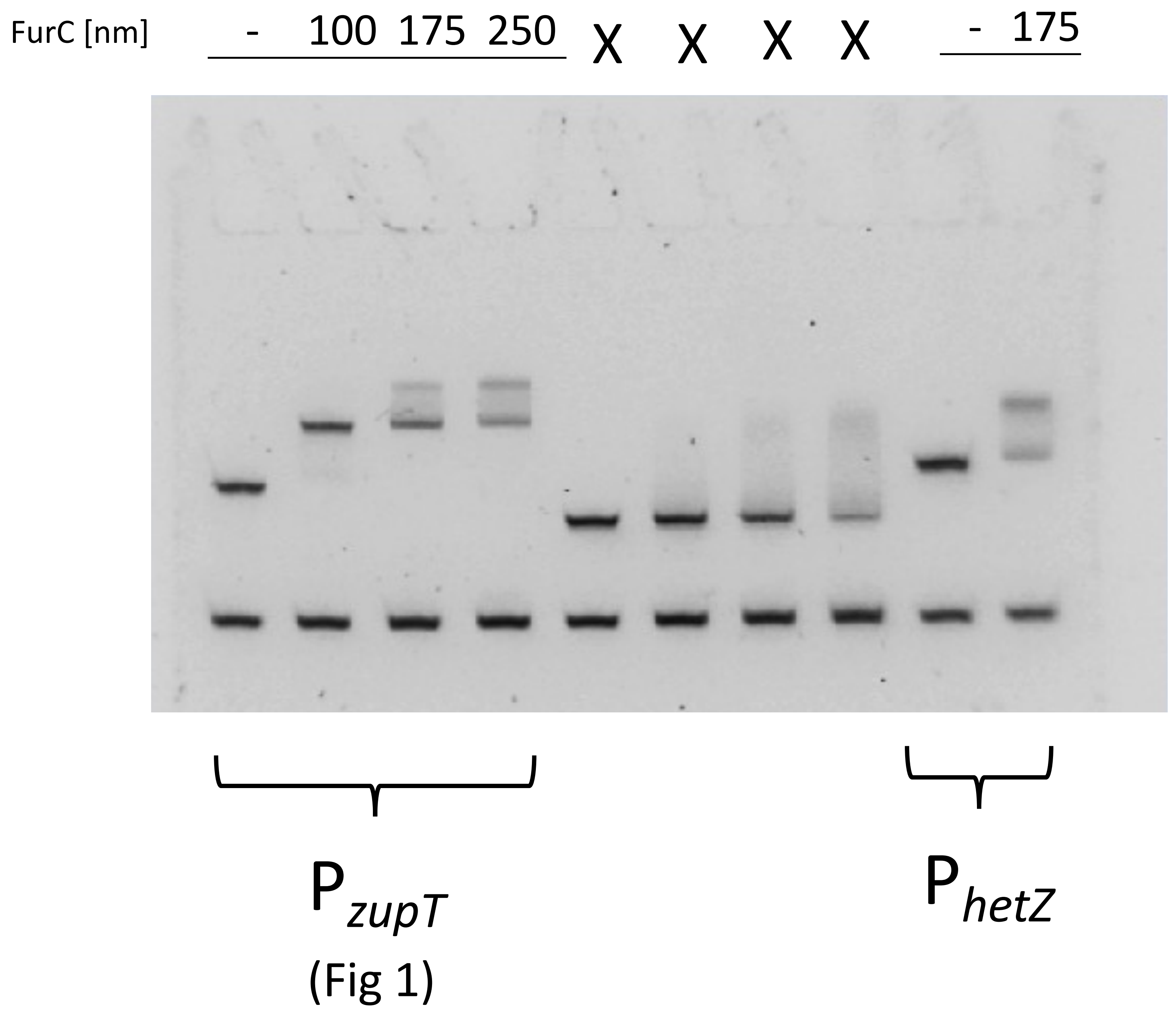
FurC [nm]
- 100 175 250
X X X X
- 175
P zupT
(Fig 1)
P hetZ

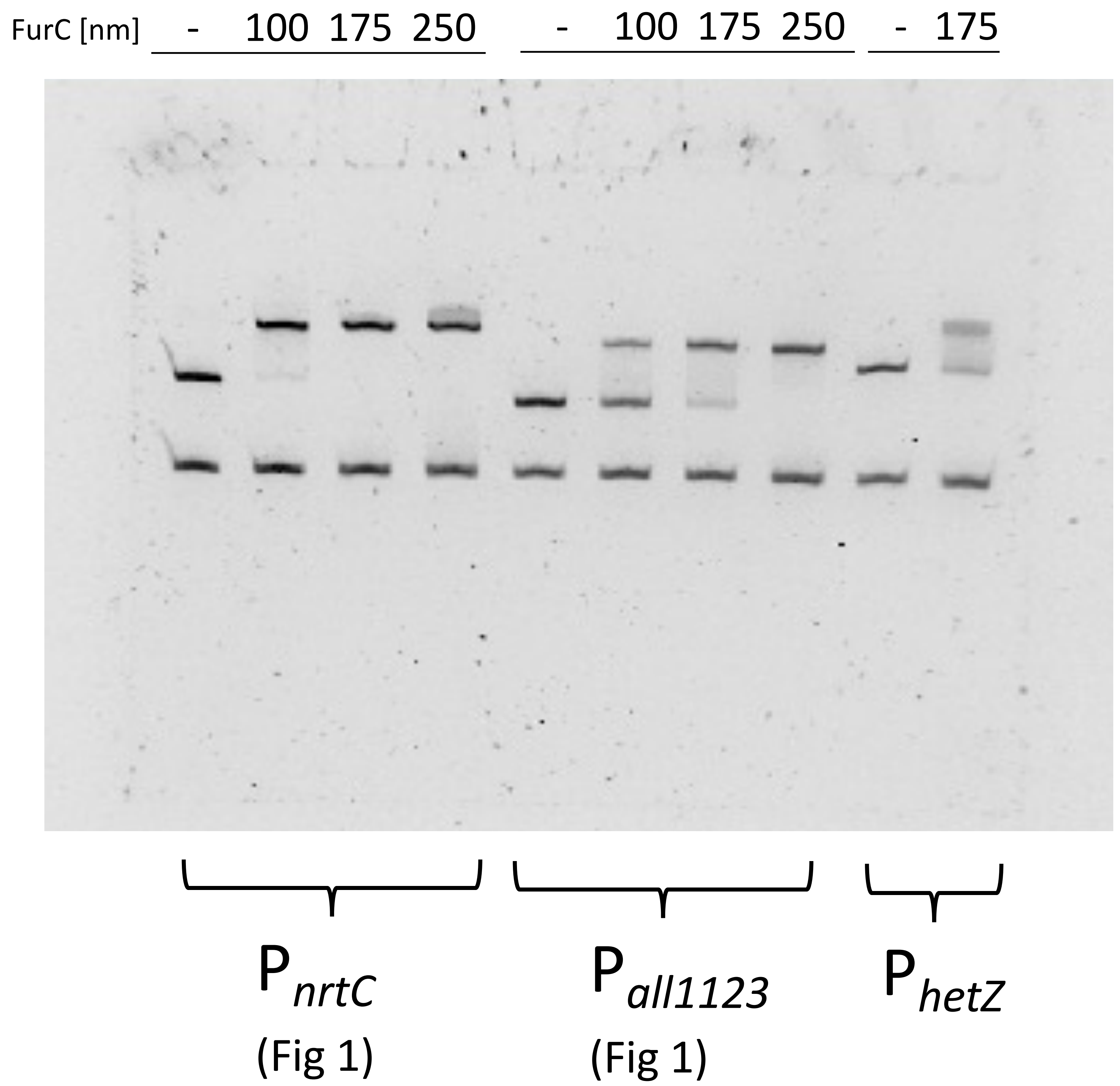
FurC [nm]
- 100 175 250
- 100 175 250
- 175
P nrtC
(Fig 1)
P all1123
(Fig 1)
P hetZ

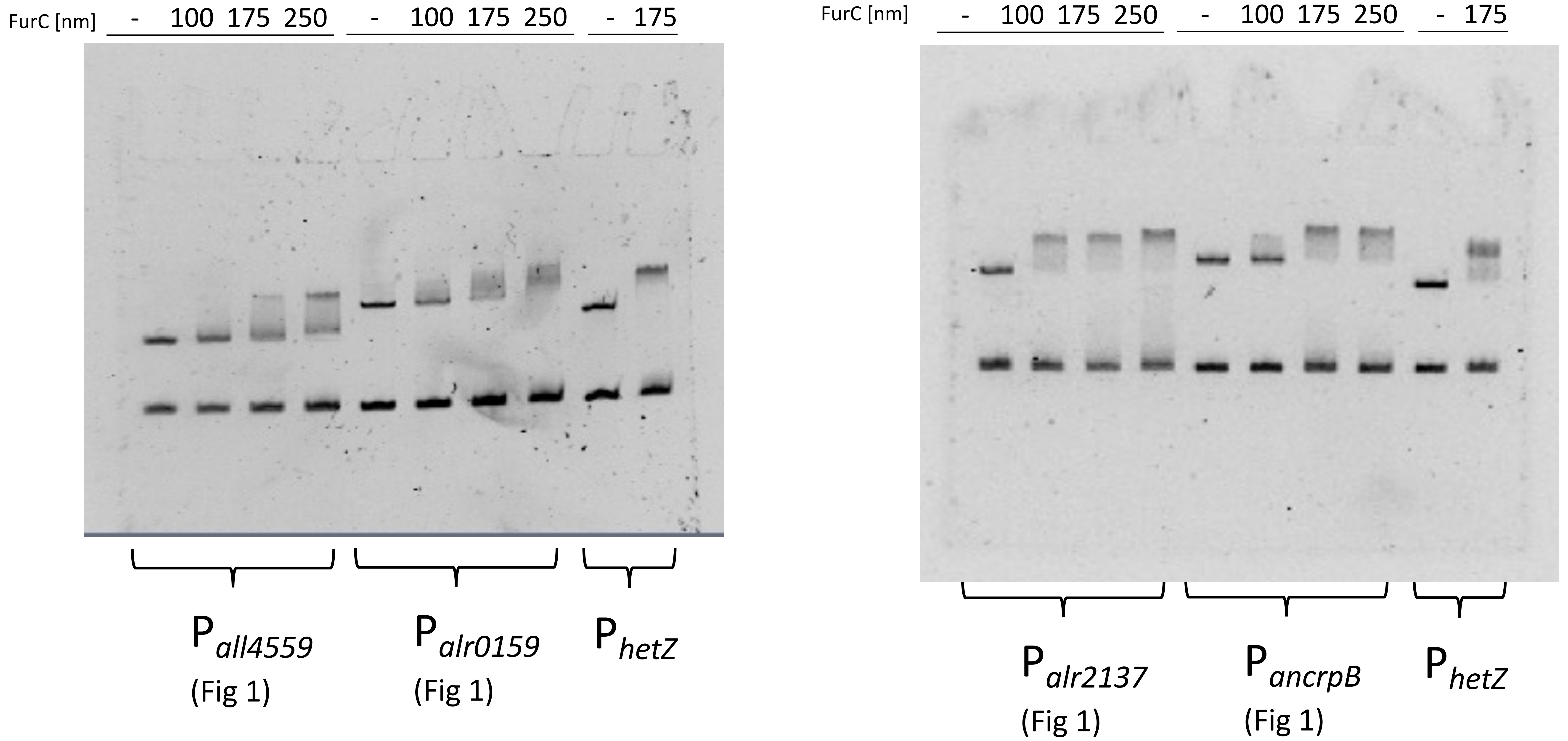
FurC [nm]
- 100 175 250
- 100 175 250
- 175
$P_{all4559}$
(Fig 1)
$P_{alr0159}$
(Fig 1)
P_{hetZ}
FurC [nm]
- 100 175 250
- 100 175 250
- 175
$P_{alr2137}$
(Fig 1)
P_{ancrpB}
(Fig 1)
P_{hetZ}

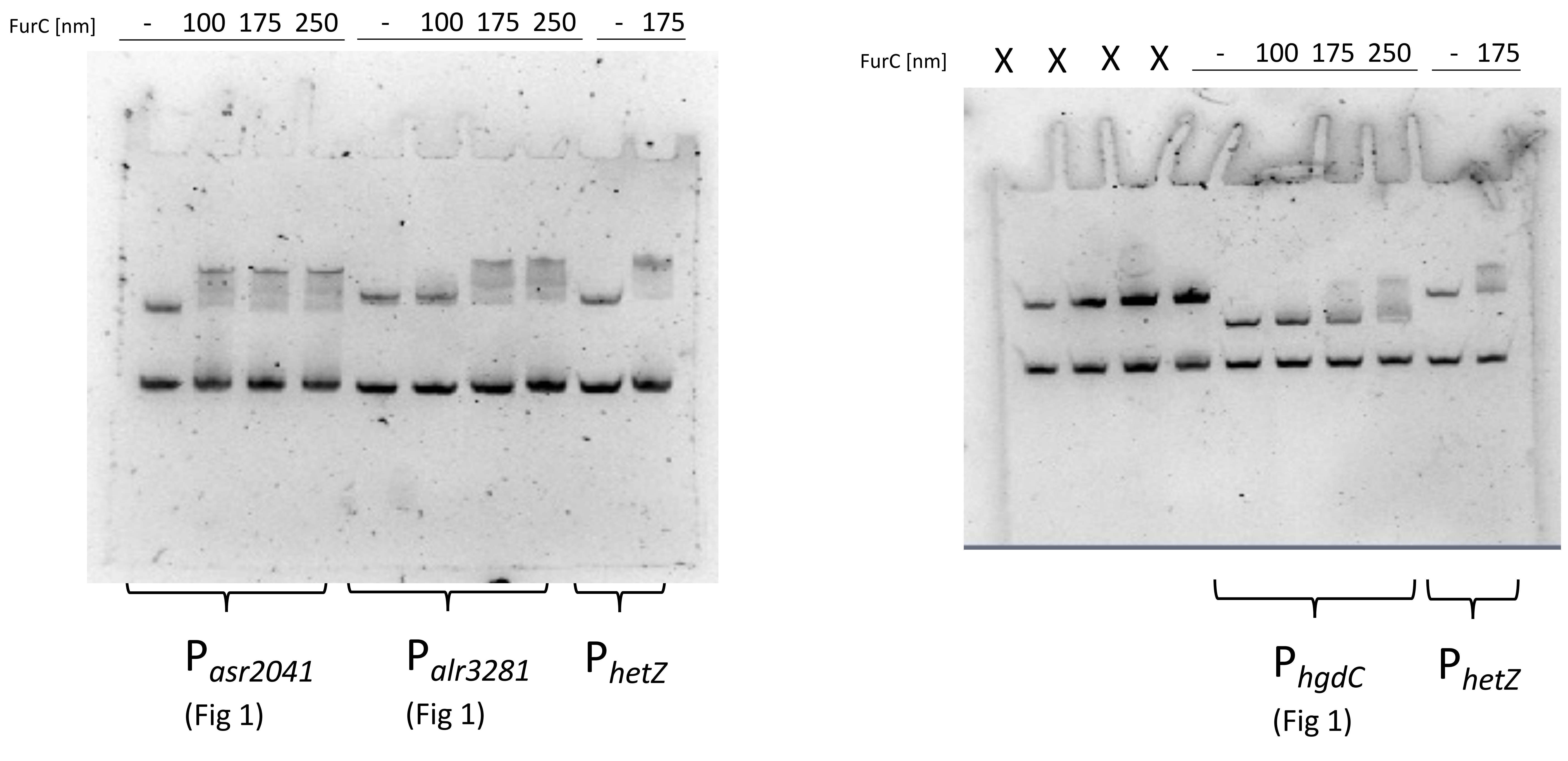
FurC [nm]
- 100 175 250
- 100 175 250
- 175
$P_{asr2041}$
(Fig 1)
$P_{alr3281}$
(Fig 1)
P_{hetZ}
FurC [nm]
X X X X
- 100 175 250
- 175
P_{hgdC}
(Fig 1)
P_{hetZ}

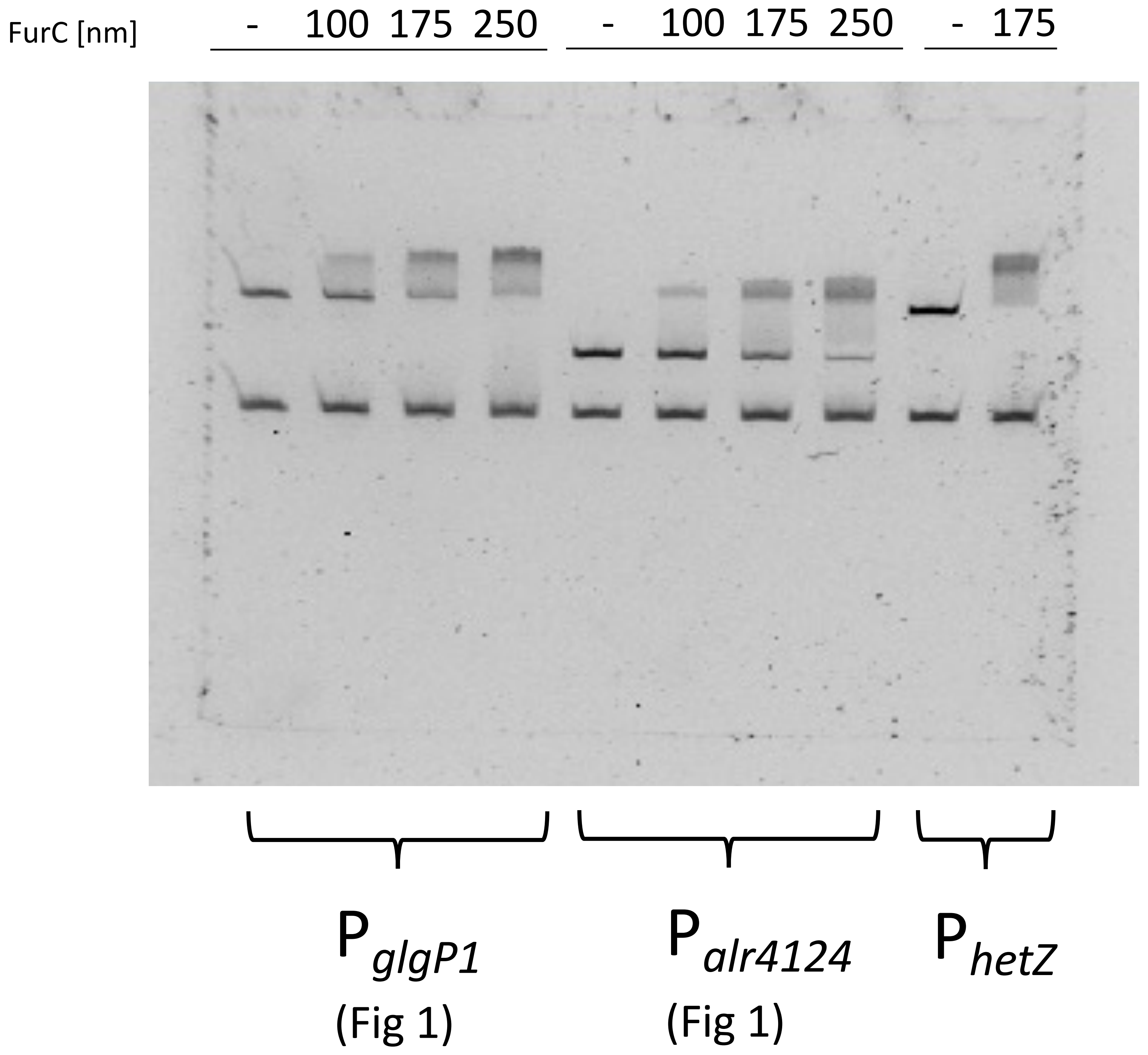
FurC [nm]
- 100 175 250 - 100 175 250 - 175
P_{glgP1}
(Fig 1)
$P_{alr4124}$
(Fig 1)
P_{hetZ}

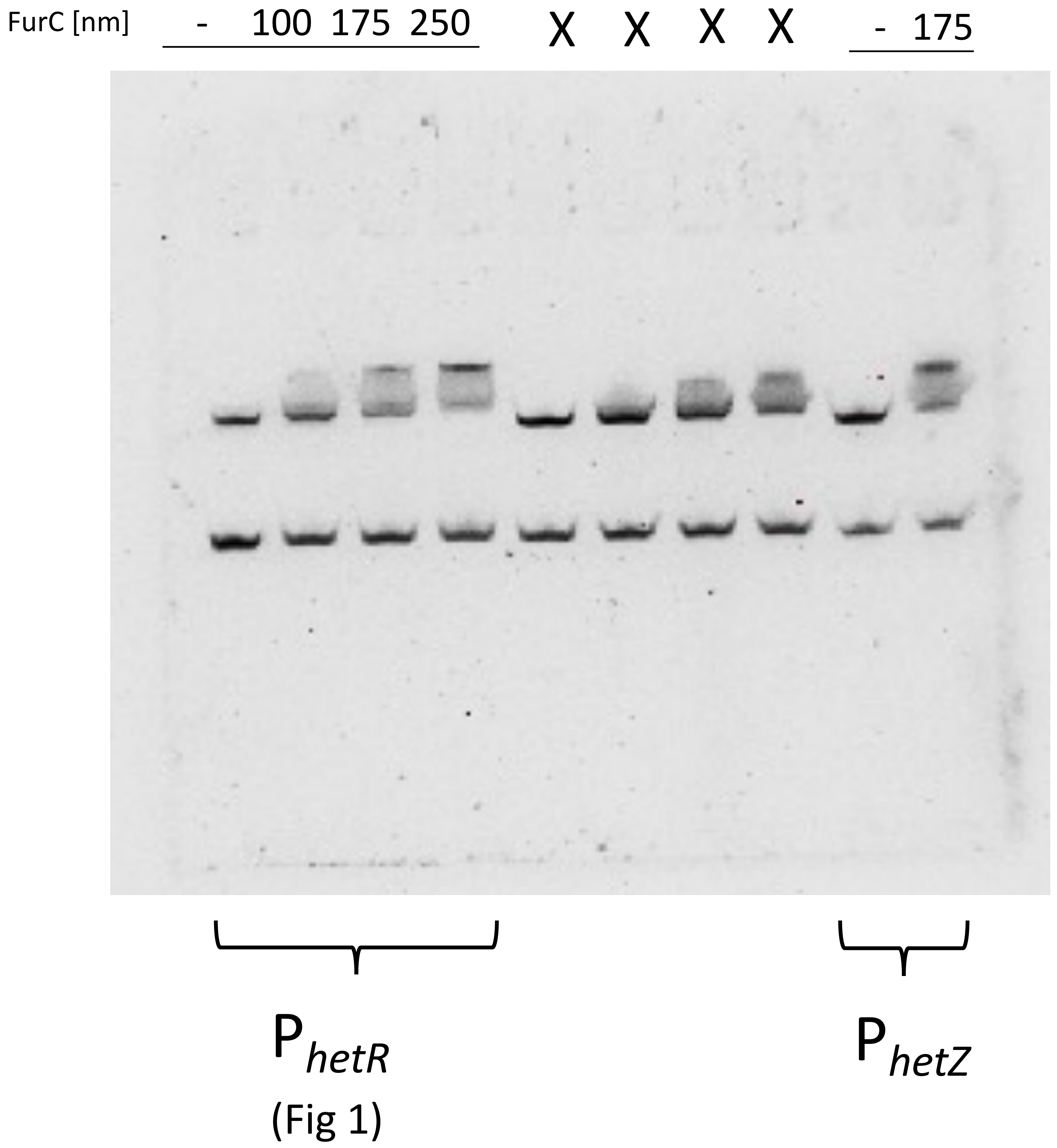
FurC [nm]
- 100 175 250 X X X X - 175
P_{hetR}
(Fig 1)
P_{hetZ}

Images of uncropped gels from EMSA results presented in Fig 4

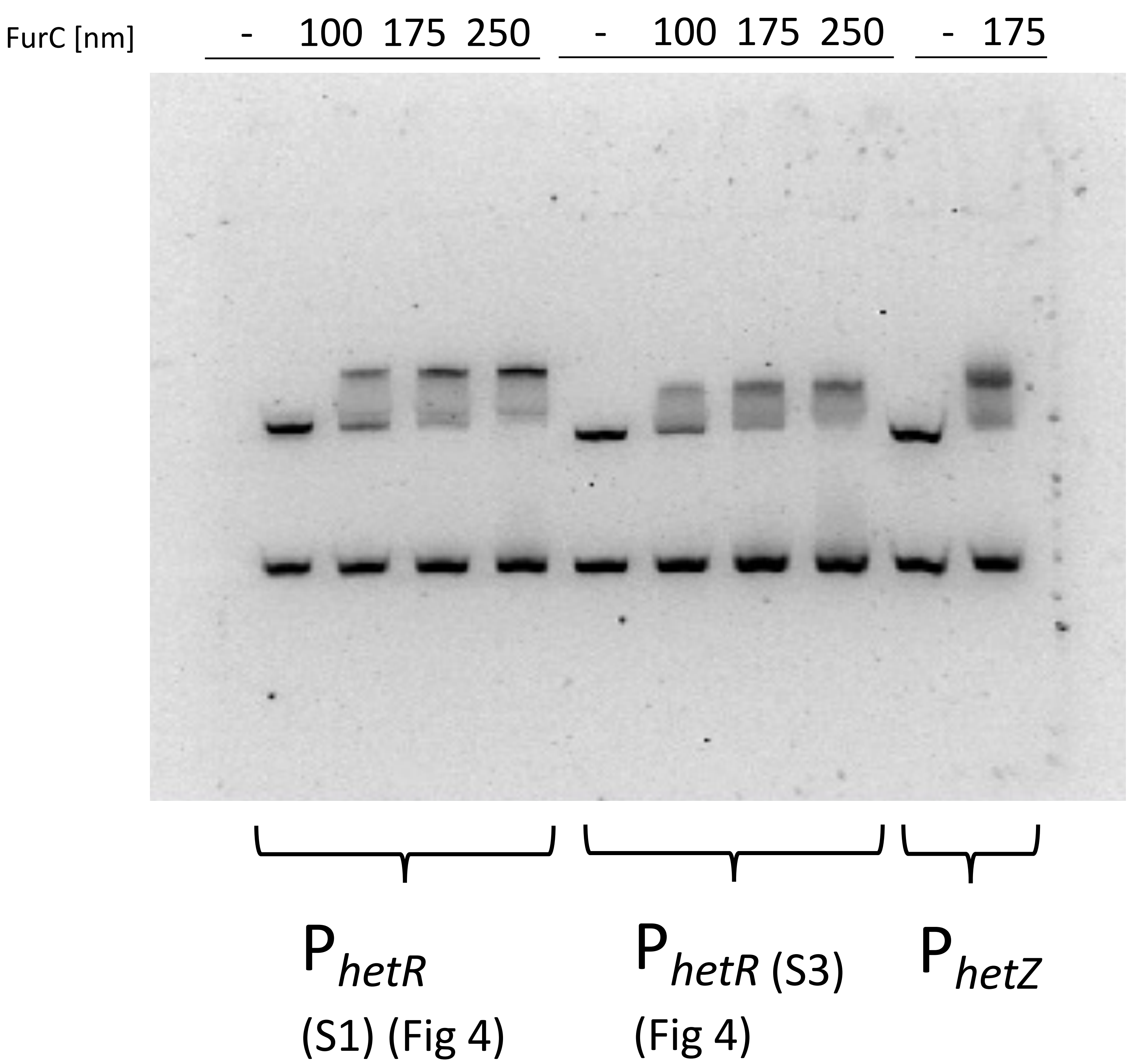
FurC [nm]
- 100 175 250 - 100 175 250 - 175
P hetR (S1) (Fig 4)
P hetR (S3) (Fig 4)
P hetZ

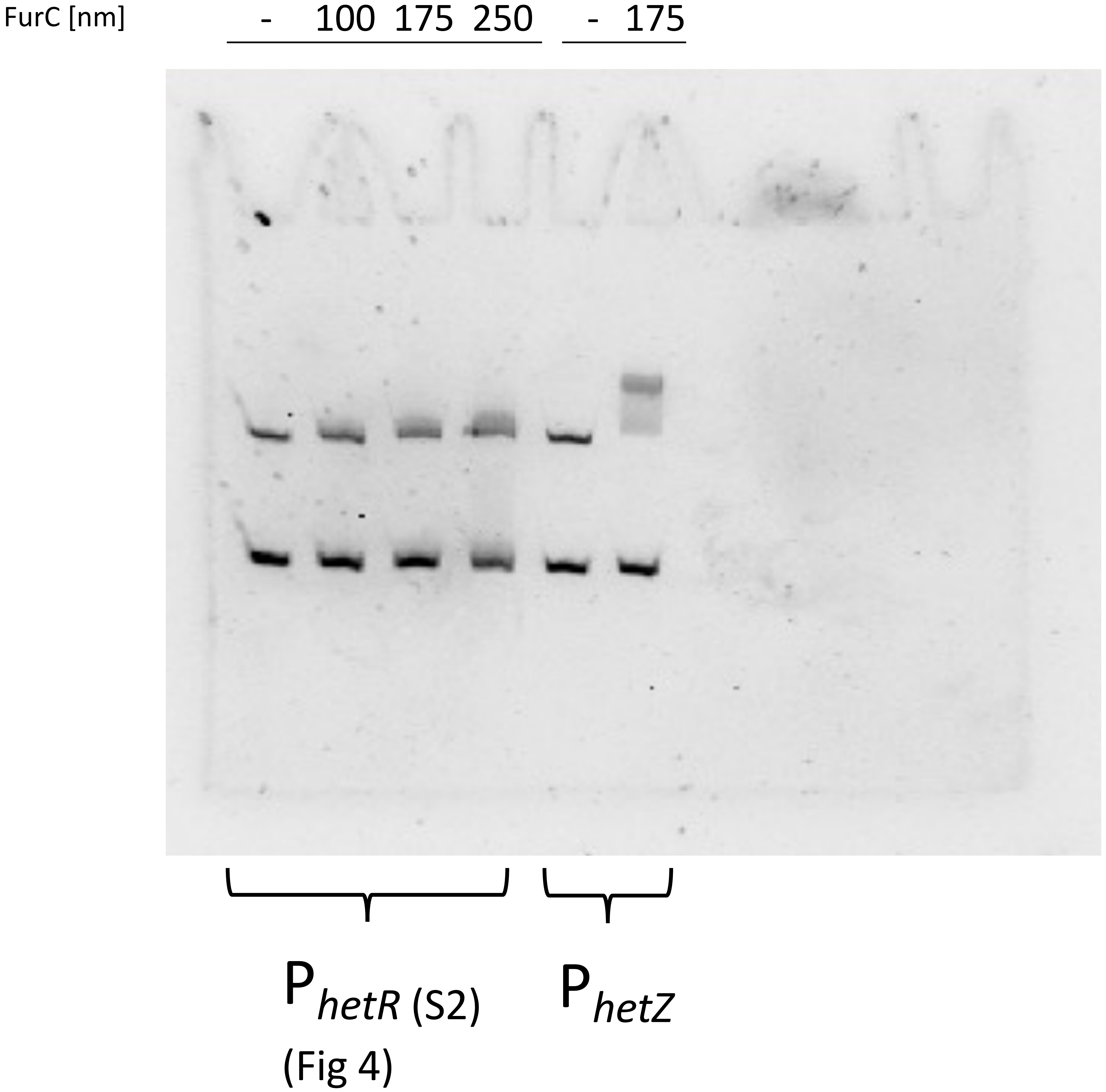
FurC [nm]
- 100 175 250 - 175
P hetR (S2) (Fig 4)
P hetZ
